# Transcriptional signatures in prefrontal cortex confer vulnerability versus resilience to food and cocaine addiction-like behavior

**DOI:** 10.1038/s41598-021-88363-9

**Published:** 2021-04-27

**Authors:** Mohit Navandar, Elena Martín-García, Rafael Maldonado, Beat Lutz, Susanne Gerber, Inigo Ruiz de Azua

**Affiliations:** 1 Institute for Human Genetics, University Medical Center of the Johannes Gutenberg University Mainz, Mainz, Germany; 2 Laboratory of Neuropharmacology-Neurophar, Department of Experimental and Health Sciences, Universitat Pompeu Fabra (UPF), Barcelona, Spain; 3 Hospital del Mar Medical Research Institute (IMIM), Barcelona, Spain; 4 Institute of Physiological Chemistry, University Medical Center of the Johannes Gutenberg University Mainz, Mainz, Germany; 5 Leibniz Institute for Resilience Research (LIR), Mainz, Germany

**Keywords:** Data processing, Addiction

## Abstract

Addiction is a chronic relapsing brain disease characterized by compulsive reward-seeking despite harmful consequences. The mechanisms underlying addiction are orchestrated by transcriptional reprogramming in the reward system of vulnerable subjects. This study aims at revealing gene expression alterations across different types of addiction. We analyzed publicly available transcriptome datasets of the prefrontal cortex (PFC) from a palatable food and a cocaine addiction study. We found 56 common genes upregulated in the PFC of addicted mice in these two studies, whereas most of the differentially expressed genes were exclusively linked to either palatable food or cocaine addiction. Gene ontology analysis of shared genes revealed that these genes contribute to learning and memory, dopaminergic synaptic transmission, and histone phosphorylation. Network analysis of shared genes revealed a protein–protein interaction node among the G protein-coupled receptors (Drd2, Drd1, Adora2a, Gpr6, Gpr88) and downstream targets of the cAMP signaling pathway (Ppp1rb1, Rgs9, Pde10a) as a core network in addiction. Upon extending the analysis to a cell-type specific level, some of these common molecular players were selectively expressed in excitatory neurons, oligodendrocytes, and endothelial cells. Overall, computational analysis of publicly available whole transcriptome datasets provides new insights into the molecular basis of addiction-like behaviors in PFC.

## Introduction

Addiction is defined by a chronically relapsing disorder characterized by the compulsion to seek for stimuli and to take drugs, and by the loss of control in the intake^1^. Addiction is driven, among others, by interindividual responses to genetic, epigenetic, and environmental factors determining the disease’s vulnerability or resilience. Therefore, it is critical to understand the neurobiological differences between recreational and controlled use and the loss of control and compulsive intake of the rewarding stimuli, which all are driven by transcriptional reprogramming in the brain reward system.

The neuronal circuits regulating reward and motivational behaviors involve primarily the mesocorticolimbic dopamine system, including the ventral tegmental area, nucleus accumbens (NAc), dorsal striatum, ventral pallidum, hippocampus, prefrontal cortex (PFC), and amygdala^2^. The PFC involves different brain regions (such as anterior cingulate, prelimbic, and infralimbic cortex), regulating cognitive and executive functions, including awareness, decision-making, self-control, and salience attribution. In fact, due to its dense projections to subcortical regions, the PFC exerts a top-down inhibitory control of appetitive and aversive behaviors^3–5^. Notably, neuroimaging studies in addicted subjects showed that impaired self-control is driven by reduced brain network activity, including PFC and striatum^6^.

Therefore, the chronic exposure to the reward triggers adaptations in the brain reward system, leading to the development of addiction in vulnerable individuals. Operant conditioning models in rodents have been fundamental in understanding the mechanisms involved in addiction. These self-administration models have been extensively used to measure the positive reinforcing effects of stimuli and reward effectiveness^2^ and addiction-like behaviors promoted, e.g., by palatable food and cocaine^5, 7–9^. Neuronal adaptations associated with addiction-like behaviors are driven by reprogramming of gene expression. Thus, numerous gene expression studies have been carried out to reveal molecular players underlying addiction in self-administration models using either drugs of abuse or natural rewards^5, 8–12^. However, the knowledge about core gene expression signatures is still elusive amongst the different types of addiction, despite that they elicit similar adaptations and behavioral changes^13^.

Therefore, we performed a computational analysis of the publicly available whole transcriptome datasets of the PFC from two independent self-administration studies in mice using palatable food and cocaine as reinforcers, respectively^5, 9^. Importantly, in both studies, mice were scored with addiction criteria-index based on their operant behavior, which allowed to classify them as addicted (vulnerable) and non-addicted (resilient) mice. After analyzing the datasets, 56 core genes were found to be upregulated in both addiction-like conditions. Gene ontology analysis of common genes revealed biological processes associated with addiction. Protein–protein association analysis identified a hub network of several shared genes at the protein level. Using single-cell RNA-seq data from a publicly available study^14^, we could allocate the shared molecular players in a cell-type-specific manner.

## Results

### Gene expression signature in PFC of addiction-like behaviors associated with palatable food and cocaine addiction

**Figure 1:**
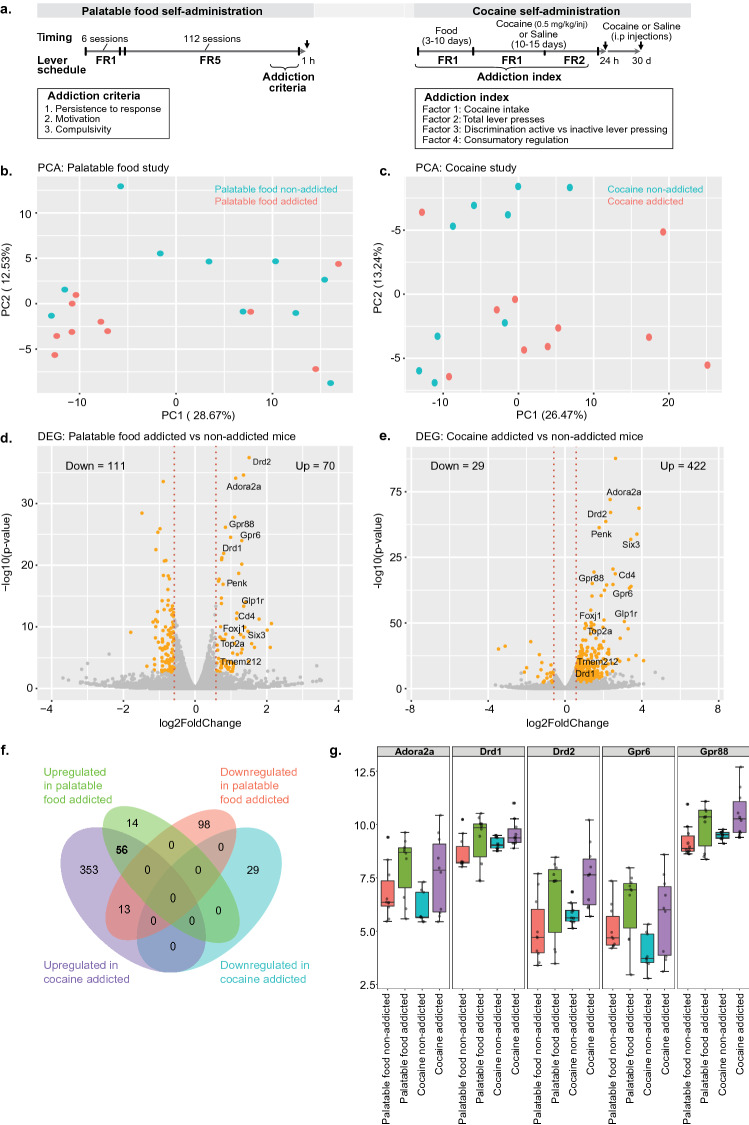
Gene expression pattern associated with food and cocaine addiction-like behaviors. (**a**) Experimental design of palatable food and cocaine self-administration studies. Black arrows indicates tissue collection in each study. (**b**) PCA plot explaining the transcriptome of palatable food non-addicted and addicted mice. (**c**) PCA plot analysis of the transcriptome of cocaine non-addicted and addicted mice. (**d**) Volcano plot of RNA-seq data representing the gene expression changes of significantly upregulated genes (70) and down-regulated genes (111) in mice addicted to palatable food as compared to mice non-addicted to palatable food. (**e**) Volcano plot of RNA-seq data representing the gene expression changes of significantly upregulated genes (422) and significantly down-regulated genes (29) in cocaine addicted mice as compared to cocaine non-addicted mice. (**f**) Venn diagram representing the overlap of differentially expressed genes from palatable food and cocaine studies. (**g**) Expression levels of Adora2a, Drd1, Drd2, Gpr6, and Gpr88 in palatable food and cocaine non-addicted and addicted mice.

We performed a computational analysis of whole transcriptomic data of PFC from palatable food addiction-like behavior^5^ and cocaine addiction^9^ studies from NCBI-GEO (Fig. 1a). For the food addiction study, mice were exposed to a self-administration model using chocolate-flavored pellets as reinforcers under a fixed ratio (FR) 1 schedule of reinforcement during six sessions. Then, FR5 schedule across 112 sessions followed to mimic the transition to addiction by the repeated seeking of palatable food (Fig. 1a). After the extended operant conditioning training, mice were classified into addicted and non-addicted mice according to three addiction criteria^5^ (Fig. 1a). In the cocaine study, mice were food trained, followed by a cocaine or saline self-administration paradigm under FR1 and FR2 schedule across 10–15 days (Fig. 1a). Notably, an addiction index was assigned to each mouse based on its operant behavior in the self-administration model^9^ (Fig. 1a). In the cocaine self-administration study, we focused on those samples with the lowest (n = 10) and highest (n = 10) addiction index in the cocaine self-administration group, independently whether the mice were challenged with cocaine withdrawal or cocaine/context priming before sample collection, thereby resembling the classification criteria of the palatable food addiction study. Thus, we compared the whole transcriptomic data of those mice with the lowest and the highest addictive-like criteria/index in both studies. Upon performing the clustering analysis, we could determine the transcriptional variation between palatable food and cocaine-addicted vs. non-addicted mice, respectively (Fig. 1b,c), confirming the behavioral characterization on the addiction-like criteria/index. The principal component analysis (PCA) showed that the transcriptome strongly changed on the PC1 and PC2, allocating the mice into two clusters, indicating that differences in gene expression led to behavioral changes. Next, using a differential expression analysis we found 111 down-regulated and 70 upregulated genes between palatable food non-addicted and addicted mice (Fig. 1d; Tables 1, 2, Supplementary Table 1), while 29 genes were down-regulated and 422 genes were upregulated between cocaine non-addicted and addicted mice (Fig. 1e; Tables 1, 2, Supplementary Table 2). Interestingly, 56 upregulated genes were associated with both palatable food and cocaine addictive-like behaviors, whereas an overlap of 13 genes were up-regulated in the cocaine-addicted mice, but down-regulated in palatable food addicted mice (Fig. 1f; Tables 1 and 2). Dopamine D2 receptor (Drd2), adenosine 2A receptor (Adora2a), G protein-coupled receptor 88 (Gpr88), dopamine D1 receptor (Drd1), G protein-coupled receptor 6 (Gpr6), Glucagon-like peptide 1 receptor (Glp1r), Galanin type-1 receptor (Galr1), proenkephalin (Penk), choline O-acetyltransferase (Chat) and regulator of G protein signaling 9 (Rgs9) were upregulated in both studies, indicating the strong link with addiction-like behaviors in the PFC (Fig. 1g; Supplementary Fig. 1a; Table 1). Additionally, some transcription factors, such as forkhead box J1 (Foxj1), Isl LIM/homeobox 1 (Isl1), sine oculis related homeobox 3 (Six3), transcriptional activator Myb (Myb), and PR domain containing 12 (Prdm12) were also differentially expressed between the addicted and non-addicted-like conditions in both studies (Supplementary Fig. 1b; Table 1). Furthermore, our analysis could identify a unique gene expression pattern for palatable food and cocaine addiction (Fig. 1f, Supplementary Tables 1, 2). Thus, 14 upregulated and 98 downregulated differentially expressed genes were particularly associated with food addiction, whereas 353 upregulated and 29 downregulated genes were found specifically in cocaine-addicted mice as compared to cocaine non-addicted mice (Fig. 1f, Supplementary Tables 1, 2), suggesting that most of the transcriptional reprogramming was exclusively associated with either palatable food or cocaine. Thus, our analysis identified a common and unique gene signature in the PFC linked to addiction-like behaviors.

**Table 1:** List of common upregulated genes in palatable food and cocaine addicted mice.

Shared genes	Palatable food study					Cocaine study				
	Non-addiction (mean reads)	Addicted (mean reads)	log 2 fold change	p-value	padj	Non-addiction (mean reads)	Addicted (mean reads)	log 2 fold change	p-value	padj
Ankub1	21.84	33.10	0.60	0.001248	0.058955	31.40	57.41	0.87	9.61E − 06	0.000583
Gpr101	33.73	51.43	0.61	7.37E − 05	0.005809	20.94	33.99	0.70	0.002161	0.056409
4932418E24Rik	56.85	87.98	0.63	6.01E − 07	8.31E − 05	14.34	45.18	1.66	1.23E − 12	3.01E − 10
Pde10a	2323.67	3646.99	0.65	3.85E − 18	3.46E − 15	1960.02	4117.73	1.07	1.64E − 25	1.39E − 22
Spint1	35.14	55.36	0.66	1.33E − 05	0.001369	46.50	73.16	0.65	0.00015	0.006242
Penk	783.63	1248.17	0.67	1.88E − 18	1.76E − 15	671.88	2303.16	1.78	4.68E − 62	1.59E − 58
Foxj1	73.18	118.38	0.69	4.57E − 09	1.17E − 06	107.67	276.29	1.36	6.92E − 26	6.12E − 23
Lrrc74b	14.43	23.37	0.70	0.002005	0.08535	23.77	48.96	1.04	4.72E − 07	4.17E − 05
Mapk15	17.45	28.55	0.71	0.000452	0.025996	45.56	79.84	0.81	6.89E − 07	5.81E − 05
Thbs4	94.63	155.02	0.71	9.42E − 11	3.51E − 08	188.21	477.06	1.34	1.32E − 30	1.34E − 27
Ttc21a	24.79	40.92	0.72	4.2E − 05	0.003615	29.07	75.10	1.37	1.11E − 13	3.17E − 11
Dmkn	18.43	30.60	0.73	0.000159	0.010971	11.35	34.70	1.61	1.76E − 10	3.15E − 08
Prkcd	205.46	341.54	0.73	2.1E − 15	1.68E − 12	236.57	626.57	1.41	5.53E − 36	5.92E − 33
Cdhr3	16.90	28.15	0.74	0.000412	0.02409	24.36	56.50	1.21	2.32E − 09	3.3E − 07
Rgs9	885.90	1478.58	0.74	1.26E − 21	1.81E − 18	1029.04	2103.36	1.03	1.23E − 23	8.63E − 21
Ppp1r1b	1067.42	1790.87	0.75	6.36E − 22	9.81E − 19	1417.70	2698.42	0.93	3.47E − 19	1.91E − 16
Serpina9	48.40	81.38	0.75	1.01E − 08	2.25E − 06	8.67	92.99	3.42	1.39E − 57	3.53E − 54
Magel2	19.46	33.00	0.76	8.95E − 05	0.0068	75.29	134.84	0.84	3.81E − 09	5.19E − 07
Spag16	20.57	35.56	0.79	2.28E − 05	0.002163	13.82	30.39	1.14	1.04E − 05	0.000618
Drd1	459.60	798.51	0.80	1.22E − 22	2.02E − 19	553.74	847.85	0.61	1.5E − 08	1.74E − 06
Top2a	38.44	67.88	0.82	1.12E − 08	2.44E − 06	57.25	162.06	1.50	4.47E − 25	3.63E − 22
Gpr88	687.07	1234.93	0.85	7.12E − 27	2.2E − 23	727.44	1946.91	1.42	8.86E − 41	1.5E − 37
Clic6	37.33	67.53	0.86	1.5E − 09	4.37E − 07	16.57	101.70	2.62	1.87E − 44	3.47E − 41
Lrp2	15.82	28.67	0.86	3.37E − 05	0.003035	6.65	30.57	2.20	3.34E − 14	1.08E − 11
Npffr1	12.30	22.48	0.87	0.000135	0.009712	8.56	23.89	1.48	3.21E − 07	3.04E − 05
Tmem212	9.08	17.53	0.95	0.000385	0.022814	16.63	56.32	1.76	6.11E − 16	2.44E − 13
Clspn	11.08	21.68	0.97	4.45E − 05	0.003739	12.07	33.78	1.48	7.35E − 09	9.58E − 07
Lrrc10b	142.24	282.65	0.99	3.03E − 25	6.54E − 22	62.90	389.65	2.63	1.97E − 88	4.02E − 84
Galr1	6.02	12.02	1.00	0.001666	0.074447	2.92	10.22	1.81	1E − 04	0.004421
Ak7	13.11	27.10	1.05	1.74E − 06	0.000222	30.52	93.19	1.61	7.26E − 20	4.22E − 17
Isl1	18.45	38.22	1.05	5.41E − 09	1.33E − 06	6.83	32.99	2.27	1.16E − 15	4.43E − 13
Myb	7.13	14.91	1.06	0.00027	0.017218	13.80	34.27	1.31	1.78E − 07	1.81E − 05
Ido1	7.40	15.65	1.08	9.76E − 05	0.007287	5.78	28.07	2.28	7.38E − 14	2.21E − 11
Tacr3	14.57	31.15	1.10	5E − 08	9.47E − 06	15.21	26.95	0.82	0.001561	0.044583
Syndig1l	113.16	243.70	1.11	1.6E − 28	5.77E − 25	102.68	447.41	2.12	2.1E − 64	8.54E − 61
Adora2a	168.79	369.92	1.13	7.78E − 35	5.6E − 31	74.89	381.74	2.35	9.45E − 73	9.61E − 69
Dlk1	23.52	52.47	1.16	4.78E − 12	2.29E − 09	172.22	258.57	0.59	6.7E − 07	5.68E − 05
Cd4	24.91	55.89	1.17	5.74E − 13	3.02E − 10	21.88	123.46	2.50	2.89E − 46	6.53E − 43
Prdm12	9.18	20.78	1.18	2.94E − 06	0.000349	5.05	54.69	3.44	1.17E − 39	1.59E − 36
Slc5a7	39.33	91.36	1.22	2.13E − 19	2.42E-16	36.94	84.27	1.19	8.29E − 12	1.87E − 09
Six3os1	12.25	29.58	1.27	1.76E − 09	5E − 07	3.27	31.91	3.29	1.64E − 23	1.11E − 20
Sh3rf2	34.41	84.86	1.30	6.75E − 21	8.09E − 18	7.07	101.83	3.85	1.91E − 69	1.3E − 65
Gpr6	45.40	111.95	1.30	1.03E − 24	2.02E − 21	17.90	101.01	2.50	2.21E − 40	3.46E − 37
Ccdc180	8.33	20.67	1.31	4.14E − 07	6.03E − 05	10.88	31.47	1.53	1.26E − 08	1.49E − 06
Ecel1	77.64	197.97	1.35	2.43E − 35	2.62E − 31	85.46	165.11	0.95	2.11E − 11	4.3E − 09
Six3	9.84	25.17	1.35	4.57E − 09	1.17E − 06	6.40	85.56	3.74	1.43E − 59	4.15E − 56
Glp1r	18.56	47.50	1.36	4.5E − 14	2.78E − 11	4.59	39.01	3.09	2.8E − 26	2.59E − 23
Ccdc153	12.15	31.77	1.39	1.19E − 10	4.2E − 08	21.63	88.32	2.03	7.75E − 27	7.5E − 24
Chat	17.07	45.10	1.40	1E − 14	7.22E − 12	4.27	31.27	2.87	7.11E − 20	4.22E − 17
Slc10a4	8.88	24.69	1.48	5.42E − 10	1.72E − 07	6.12	29.48	2.27	1.33E − 14	4.57E − 12
Spata18	3.75	10.57	1.50	4.45E − 05	0.003739	6.04	26.49	2.13	1.45E − 11	3.09E − 09
Drd2	56.60	160.87	1.51	3.58E − 38	7.74E − 34	59.07	305.98	2.37	7.48E − 68	3.8E − 64
Ankk1	5.69	16.99	1.58	5.24E − 08	9.72E − 06	2.12	15.32	2.85	4.03E − 11	8.05E − 09
Fam216b	4.64	14.62	1.66	2.15E − 07	3.36E − 05	14.79	53.67	1.86	3.06E − 17	1.48E − 14
Ntrk1	6.54	22.53	1.78	5.51E − 12	2.58E − 09	1.39	13.79	3.31	9.46E − 12	2.09E − 09
Gpx6	3.73	16.31	2.13	2.89E − 11	1.18E − 08	0.66	11.25	4.09	2.21E − 11	4.45E − 09

**Table 2:** List of shared genes downregulated in food addicted mice and upregulated in cocaine addicted mice.

Shared genes	Palatable food study					Cocaine study				
	Non-addicted (mean reads)	Addicted (mean reads)	log2 fold change	p-value	padj	Non-addiction (mean reads)	Addicted (mean reads)	log2 fold change	p-value	padj
Myoc	316.27	207.13	− 0.61	4.82E − 11	1.89E − 08	202.79	306.45	0.60	4.14E − 07	3.79E − 05
Itih2	157.71	101.37	− 0.64	1.05E − 09	3.11E − 07	70.47	108.06	0.62	2.72E − 05	0.001421
Col3a1	129.40	84.44	− 0.62	2.67E − 08	5.35E − 06	34.59	53.80	0.64	0.001594	0.045203
H2-Eb1	55.96	26.10	− 1.10	7.17E − 12	3.29E − 09	32.51	51.04	0.65	0.00066	0.021558
Slc13a4	339.80	172.47	− 0.98	1.24E − 26	3.35E − 23	93.99	148.51	0.66	1.57E − 06	0.000118
Aebp1	511.15	290.77	− 0.81	1.73E − 21	2.33E − 18	198.05	323.59	0.71	2.3E − 09	3.29E − 07
Flnc	194.79	91.30	− 1.09	3.02E − 23	5.43E − 20	37.60	62.42	0.73	4.81E − 05	0.002333
Wfikkn2	36.08	21.06	− 0.78	2.13E − 05	0.002046	12.65	22.43	0.83	0.002113	0.055372
Adamtsl3	122.07	76.07	− 0.68	4.36E − 09	1.15E − 06	16.85	31.06	0.88	0.000106	0.004613
Dct	109.34	39.13	− 1.48	3.62E − 29	1.56E − 25	14.60	27.75	0.93	0.000242	0.00935
Slc47a1	72.19	34.90	− 1.05	3.39E − 13	1.87E − 10	9.56	18.46	0.95	0.002089	0.055027
Pmel	36.86	17.12	− 1.11	6.06E − 09	1.45E − 06	8.98	17.58	0.97	0.001436	0.041895
Fgfbp1	38.73	23.74	− 0.71	2.6E − 05	0.002422	6.61	15.64	1.24	0.000334	0.012237

In order to validate our findings and the classification in the cocaine study, we performed a further analysis of the cocaine self-administrated non-addictive mice and saline self-administrated mice (Supplementary Fig. 1c). We found that some common genes (32 out of 56) were downregulated in cocaine non-addicted mice (Supplementary Fig. 1c). This analysis was only possible in the cocaine study. These results support the hypothesis of a common gene signature for addiction and suggest that downregulation of key addiction genes represents a protective mechanism underlying resilience to addiction behavior.

### Addiction signature is associated with learning and memory, dopaminergic synaptic transmission, cAMP signaling pathway, and histone phosphorylation

**Figure 2:**
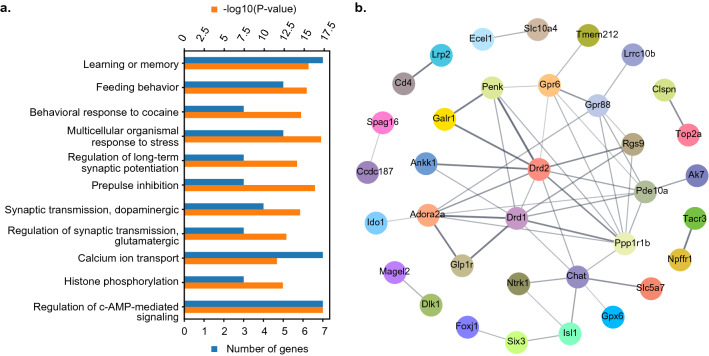
GO and gene association network analysis of shared genes in food and cocaine addiction-like behavior. (**a**) Bar plot representing the behavioral, biological, and molecular processes. Number of genes and − log 10 (p-value) in red and blue, respectively. (**b**) Protein–protein interaction network of shared genes in palatable food and cocaine addiction. The thickness of an edge represents the confidence score for the given interactions.

Gene ontology (GO) analysis of shared upregulated genes revealed that Drd2, Drd1, Ppp1r1b, Gpr88, Glp1r, and Ntrk1, among others genes, contribute to behavioral responses, including learning and memory, response to cocaine, feeding behavior, and response to stress (Fig. 2a, Supplementary Table 3). The GO analysis also showed gene’s participation (Adora2a, Drd2, Drd1, Rgs9, Ntrk1) in biological processes related to synaptic plasticity, such as long-term potentiation, prepulse inhibition, and regulation of both glutamatergic and dopaminergic synaptic transmission (Fig. 2a, Supplementary Table 3). Finally, the analysis identified gene expression changes (Adora2a, Drd2, Drd1, Ppp1r1b, Pde10a, Rgs9, CD4, Glp1r) at molecular level functions, including in the regulation of cAMP signaling pathway, calcium ion transport, and histone phosphorylation (Fig. 2a, Supplementary Table 3). Notably, genes encoding dopamine- and adenosine-mediated cAMP signaling pathway, including Drd2, Drd1, Adora2a, and the downstream target Ppp1r1b (encoding the dopamine and cAMP-regulated neuronal phosphoprotein, DARPP-32), make a strong contribution to the above analysis.

### Drd2, Adora2a, Drd1, Gpr88, and Gpr6, together with downstream targets of cAMP signaling pathway, establish a hub for a network at protein levels

The computational and GO analysis gave the basis to perform functional protein–protein association network analysis among the shared genes using the STRING database^15^. In Fig. 2b, every node represents one gene, and each edge connecting two nodes represents different degrees of associations at protein levels. The thickness of an edge visualizes the confidence score for the given interactions. The STRING analysis revealed a protein–protein interaction network of 35 shared genes. Out of these candidates, eight genes, five encoding G-protein-coupled receptors (GPCRs, including dopamine D2 receptor (D2); dopamine D1 receptor (D1); adenosine 2A receptor (A2A); Gpr88 receptor (GPR88), and Gpr6 receptor (GPR6)) are building the core of this network, together with three further proteins, all related to the cAMP signaling pathway (DARPP32; phosphodiesterase 10a or PDE10A, encoded by phosphodiesterase 10a (Pde10a) gene; and a regulator of G-protein signaling-9 or RGS9, encoded by Rgs9 gene (Fig. 2b). Accordingly, previous studies showed that the formation of D1–D2 heteromers modifies the functional properties of these receptors by coupling to Gq proteins and increasing the sensitivity to amphetamine^16^. Functional D2–A2A heteromers have also been recently demonstrated^17^.

Furthermore, synergistic interaction between Drd2 and Adora2a genes might play a role in anxiety disorders^18^. The co-occurrence between anxiety disorders and substance abuse disorders has a higher prevalence than expected by chance level^19^. Thus, previous data validate the protein–protein interaction network analysis of gene signature of PFC in addiction-like behaviors. We propose that this gene network's core might make the most considerable contribution to the neuroplasticity and adaptation associated with addiction-like behaviors.

### Cell type-specific expression of the shared molecular players

**Figure 3:**
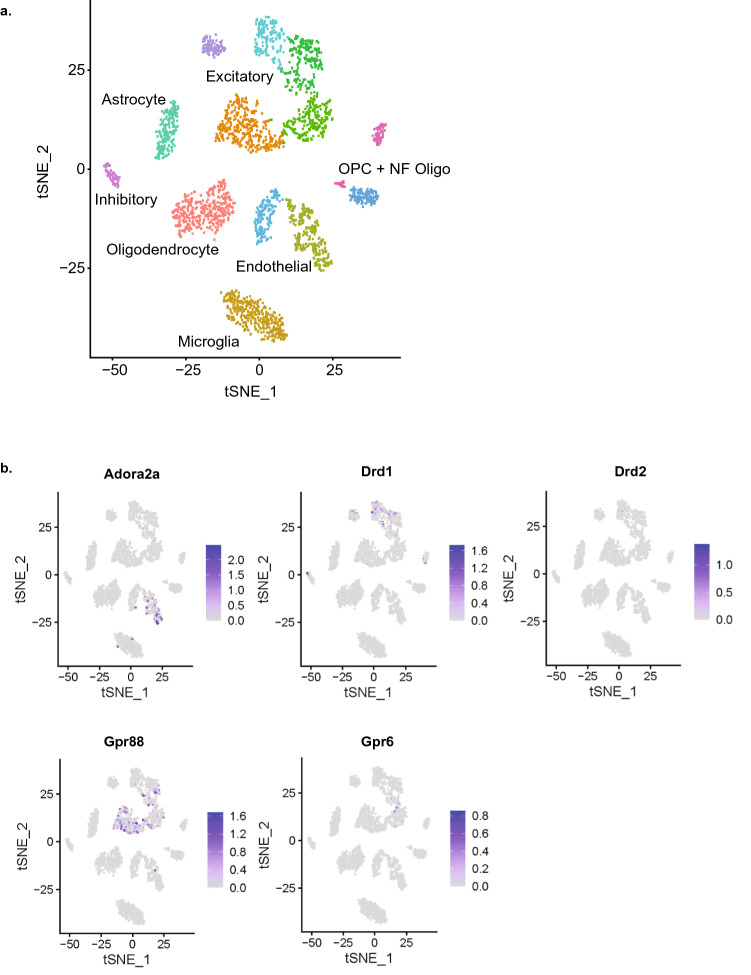
Cell type specific expression of the relevant genes in mouse PFC. (**a**) t-SNE plot representing different cell type clusters in PFC. t-SNE plot representing the clusters of the different cell subtypes in PFC based on the transcriptome of cocaine self-administration mice during the habituation phase from Bhattacherjee et al.^14^. (**b**) t-SNE plot representing some of the common upregulated genes found in cocaine and food addiction studies.

Finally, we investigated the cell type selective expression of the shared genes using the publicly available single-cell RNA-seq data of the PFC (including anterior cingular, prelimbic, and infralimbic cortex) in adult male C57BL/6 mice^14^. In the present study, we focused on the dataset of cocaine self-administration mice during the maintenance phase to study the impact of long-term exposure to cocaine on transcriptional changes at cell type specific levels. First, we determined the different cell subtypes stated in Bhattacherjee et al.^14^ using tSNE approach and replicated this (Fig. 3a). After visualizing the specific markers for PFC cell clusters (Supplementary Fig. 2), we could identify the expression of 28 shared genes in the different cell type clusters (Fig. 3b; Supplementary Figs. 3, 4). Drd1, Drd2, Gpr88, and Gpr6 were almost exclusively expressed in excitatory neurons. However, Gpr88 and Drd1 were also found at lower levels in inhibitory neurons and non-neuronal cells, such as oligodendrocyte precursors (OPC) and endothelial cells (Fig. 3b). Strikingly, Adora2a was mostly expressed in endothelial cells, at lesser levels in microglia, and very sparse expression was observed in excitatory and inhibitory neurons (Fig. 3b). The regulatory genes (Ppp1r1b, Rgs9, and Pde10a) of the cAMP signaling pathway showed a broad expression in the different cell clusters, including excitatory neurons, inhibitory neurons, astrocytes, oligodendrocytes, OPC, newly formed oligodendrocytes (NF oligo), and endothelial cells (Supplementary Figs. 3, 4). The transcription factor Foxj1 also showed a broad expression and was expressed in excitatory neurons, endothelial cells, oligodendrocytes, and NF oligo. However, other genes showed a very selective expression in one of the clusters, such as Cd4, Ido1, and Dmkn in excitatory neurons, Top2a in NF oligo, Slc5a7 in endothelial cells, and Spint1 in microglia (Supplementary Figs. 3, 4).

## Discussion

Addiction is defined by behavioral abnormalities, including a loss of control over reward intake, a compulsive reward intake despite aversive consequences, and chronic relapse after long periods of abstinence. Importantly, the same behavioral abnormalities associated with the addiction symptoms are driven by diverse rewarding stimuli (drug of abuse, natural rewards, and other stimulants), suggesting a common pattern of cellular adaptations in the brain reward circuit of vulnerable individuals. Despite that a huge number of studies have tried to determine the molecular basis of addiction, there is still a limited understanding of the common and unique molecular mechanisms underlying addiction disorders. In this study, we performed a computational analysis of publicly available datasets of two independent studies using animal models of palatable food and cocaine addiction^5, 9^. We uncovered a group of genes in PFC associated with vulnerability vs. resilience to addiction-like behaviors.

As a part of the brain reward system, PFC is instrumental in the control of reward intake, which is impaired in addiction leading to compulsive drug intake and relapse^3, 20^. Several human and animal model studies associated a reduced neuronal activity in PFC with compulsive behavior in reward intake^3, 5, 20, 21^. These alterations at cellular and circuitry levels are driven by transcriptional reprogramming after long-term exposure to reward intake. Accordingly, the principal component analysis of RNA-seq data in both studies showed substantial transcriptomic differences between addicted and non-addicted mice. Our computational analysis revealed that a total of 69 differentially expressed genes were found in common between these two studies. However, 13 of these genes were upregulated in cocaine-addicted mice, while the same genes were downregulated in food addicted mice, which remains to be interpreted.

Interestingly, 56 common genes were upregulated in both food and cocaine-addicted mice. These shared genes include several GPCRs (Drd2, Drd1, Adora2a, Gpr88, Gpr6, Glp1r) and transcription factors (Foxj1, Six3, Prdm12, etc.), among others. GPCRs are very attractive, as 34% of drugs approved by FDA target this receptor class^22^. Accordingly, a previous study demonstrated that D1 expressing neurons in PFC are activated by food intake, and optogenetic stimulation of these D1 neurons increased feeding^23^. Moreover, we have recently demonstrated that overexpression of Drd2 in PFC-NAc projection neurons promoted a compulsive-like behavior for chocolate pellet seeking in mice^5^. In contrast, mice with deficiency in the Gpr88 gene, which is highly expressed in the striatum and at lower levels in the cortex and thalamus of adult mice^24^, showed increased alcohol seeking and consumption^25^. However, its specific function in the PFC remains unknown. Interestingly, some preclinical studies have examined the use of GLP-1 analogs in alcohol use disorder^26^, suggesting a potential protective role or, alternatively, a compensatory mechanism of upregulated Glpr1 gene expression in PFC of vulnerable mice. A genome-wide association study identified Six3 and Drd2 loci associated with alcohol dependence^27^. Thus, the core gene signature of PFC in addiction identified by our comparative computational analysis is also supported by previous studies. Notably, the computational analysis could also identify new players associated with addiction such as transcription factors (Foxj1, Isl1, Psdm12). Hence, this study opens new avenues to be explored in future studies, for example, the role of Foxj1 and synergistic approaches targeting different GPCRs for the treatment of addiction disorders.

Besides, our analysis also identified a unique gene signature for either addiction condition. Indeed, most of the differentially expressed genes found in both studies were exclusively related to either palatable food or cocaine addiction. Interestingly, there was no convergence in the down-regulated genes associated with palatable food and cocaine addiction-like phenotype, implying that these mechanisms leading to gene expression down-regulation in PFC are distinct for each type of addiction.

Both food and cocaine, like other rewarding stimuli, increase dopamine levels in NAc, which are responsible at least in part for their reinforcing effects. Dopamine dynamics are directly mediated by the activation of dopamine neurons in the ventral tegmental area, which also sends projections to PFC, hippocampus, and amygdala apart from the mentioned NAc. Upon performing GO analysis of the shared genes, the study revealed that several genes were involved in behavioral responses, such as learning and memory, feeding behavior, response to cocaine, stress responses as well as in change in synaptic plasticity, such as long-term potentiation, prepulse inhibition, and regulation of dopaminergic and glutamatergic synaptic transmission. Reward prediction error^28^ and incentive salience^29^ hypothesis underline the importance of dopamine dynamics in brain reward areas in learning processes, as suggested by our GO analysis. Indeed, excessive learning habits have been involved in relapse and craving responses to reward-related cues previously associated with the reward intake. Recent findings have also identified that dopamine release in mPFC mediates behavioral learning responses to aversive stimuli^30^.

Likewise, the GO analysis identified a group of common genes involved in synaptic plasticity processes. Synaptic plasticity in PFC evoked by repeated exposure to the reward play a pivotal role in changes in neuronal circuits and addictive behaviors, e.g., relapse^31^. Consequently, relapse is caused by powerful and long-lasting memories of the reward experience related to synaptic plasticity changes associated with repeated reward intake.

Interestingly, histone phosphorylation was also identified by GO analysis at the molecular function level. Epigenetic mechanisms have been revealed as essential mediators of long-lasting gene expression changes linked to addiction, and stable epigenetic changes might confer addiction vulnerability^32^. Histone phosphorylation generally allows the transcription of genes and seems to play a crucial role in promoting the expression of IEG, such as c-Fos and c-Jun^33^. Several studies reported that cocaine increased phosphorylation of histone H3 in striatal neurons, and it may be important in the cocaine-induced long-term neuronal plasticity^34–36^. In this context, several compounds that inhibit histone phosphorylation are under investigation as clinical candidates in human cancer^37, 38^. Thereby, more insights will clarify the role of histone phosphorylation after chronic exposure to the reward and identify inhibitors of histone phosphorylation as a potential treatment of addiction.

The protein–protein association analysis of the shared genes showed a core network of 8 genes (Drd1, Drd2, Adora2a, Gpr88, Gpr6, Ppp1rb1b, Rgs9, Pde10), predicting protein–protein interactions at physical and at functional levels. The hub of the network includes five GPCRs (Drd1, Drd2, Adora2a, Gpr88, Gpr6) and three proteins associated with the cAMP signaling pathway (Ppp1rb1b, Rgs9, Pde10). As mentioned above, GPCRs have been investigated extensively due to their contribution to physiological and pathological processes. In this context, GPCR heterodimerization has been postulated several years ago and puts forward the concept of physical associations between two different GPCRs that might have different functional properties from those of the individual receptors^39^. Previous evidence described heterodimerization processes between dopamine D1–D2 receptors^40^ and D2–A2A receptors^17^. Recently, a BRET study has demonstrated physical interactions of GPR88-Rluc8 with mVenus-tagged D2 and mVenus-tagged A2A receptors in transfected cells^41^. However, according to our knowledge, no evidence of interaction of GPR6 with other GPCRs has been shown until now. Importantly, both D1 and A2A receptors increase cAMP levels by coupling to Gs proteins, while in contrast the, activation of D2 and GPR88 decreases cAMP levels by coupling to Gi proteins. Furthermore, RGS9 can modulate cAMP signaling by interaction with the β-subunit of the G proteins and functionally interact with D2, as suggested by previous studies^42, 43^. Moreover, the activation of cAMP signaling induces phosphorylation of DARPP-32, which regulates synaptic plasticity as well as many other biological and behavioral responses driven by drugs of abuse^44^. Finally, PDE10A selectively regulates cAMP signaling by a potentiation of A2A- and D1-mediated phosphorylation of DARPP-32, whereas it blunts D2-induced decrease in DARPP-32 phosphorylation^45^, and as a result, increases the phosphorylation of DARPP-32. In summary, the STRING database analysis revealed a protein–protein association network that disentangles the dopamine-, adenosine- and GPR88-mediated cAMP signaling pathway in PFC as a pivotal signaling pathway in addiction and identifies this signaling pathway as a potential therapeutic target in addictive disorders. Our analysis of the gene association network provides new insights to understand these psychiatric disorders and to potentially develop a new pharmacological target.

Finally, RNA-seq analysis of cell types in mouse PFC has been recently reported^14^. Single-cell RNA sequencing allows identifying transcriptional changes across different cell populations associated with physiological or pathological processes, including addiction. Bhattacherjee et al. analyzed the transcriptome dynamics in PFC cell types evoked by chronic cocaine exposure. Therefore, to better understand the cellular mechanisms involved in addiction, we asked whether our candidate genes exhibit a cell type-specific expression in PFC. Based on the fact that addiction is a chronic relapsing disorder and the role of the PFC in cognitive and executive function, we assume that the addiction-related core gene reprogramming takes place in the same cell types in PFC and remains stable across the time course of the disease. Nevertheless, we cannot exclude the possibility that palatable food-induced gene reprogramming may occur in other cell types. For this purpose, we performed computational analysis of the shared genes over the publicly available data of single-cell transcriptome in PFC of cocaine self-administration mice. We observed that some of the relevant genes have a specific expression pattern in different PFC cell clusters. Thus, Drd1, Drd2, Gpr88, Gpr6, and Rgs9 were almost exclusively expressed in excitatory neurons, although Drd1, Gpr88, and Rgs9 also showed expression in other cell clusters. Strikingly, Adora2a had a predominant expression in endothelial cells and microglia, with a very sparse expression in inhibitory and excitatory neurons. These data challenge previous evidence about the anatomical, pharmacological, and functional properties of the A2A receptor^46, 47^, although they do not entirely invalidate them. Finally, Ppp1r1b and Pde10a were broadly expressed in PFC, suggesting a role in more general cellular functions. Overall, the specific cell type expression of the addiction gene signature based on the computational analysis of a public dataset of single-cell RNA-seq in PFC suggests a significant role of the excitatory neurons in addiction and put forward those protein–protein interactions predicted by the STRING data analysis. The transcriptional study at cell-type specific levels of the addiction gene signature in PFC might be relevant to design potential new pharmaceutical approaches to tackle addiction.

In conclusion, addiction disorders share similar behavioral alterations even if they have been evoked by different rewarding stimuli. Thus, we hypothesize that chronic reward-induced neuronal plasticity is triggered by common transcriptional reprogramming to elicit addiction-like behaviors. Nevertheless, we could not discard that some of these gene expression changes are related to an interindividual predisposition that confers a particular vulnerability to addiction. This study uncovered the common and unique differentially expressed genes in PFC in addiction by computational analysis of public RNA-seq datasets from two independent studies using palatable food and cocaine addiction animal models. Thus, we identified 56 shared genes present in addicted mice as a gene expression signature of addiction in the PFC. These genes contribute to learning and memory responses, synaptic plasticity processes, and regulation of cAMP signaling pathway as suggested by GO analysis. Furthermore, protein–protein association analysis of the candidate genes identified a core network consisting of dopamine, adenosine and orphan GPR88-mediated G protein-coupled cAMP signaling pathway as key players of neuroplasticity changes in PFC during addiction. Finally, computational analysis of public single-cell RNA-seq data suggests that transcriptional reprogramming of the relevant genes in PFC occurred mainly in excitatory neurons. This study unravels a common and unique gene expression signature of PFC that confers the vulnerability and resilience to addiction and disentangle a core network of eight genes that may pave new avenues to develop pharmacological treatments that alleviate the chronic relapse and the compulsivity associated with the addiction syndrome.

## Methods

### Data collection

Transcriptomics data were obtained from the NCBI-GEO. For current study we considered GSE139482, GSE110344 and GSE124952. Transcriptomics data was received as raw files in fastq format from EBI.

### RNA-sequencing data analysis (quality check, alignment, normalization and differential gene expression analysis)

After receiving raw data in fastq format, quality of individual sample was checked using FASTQC version v0.10.5. Sample passing the quality were subjected for the alignment using TopHat^48^ version v2.153 to the mouse genome (mm9) with default parameters. Mapped reads were considered for read count per gene using HTSeq^49^ version 0.954. Output of HTSeq (read counts per gene) was normalized and differential gene expression analysis was performed using R package DESeq with false discovery rate (FDR) rate of 0.1. “plotPCA” function from DEseq^50^ package was used to check variability between the non-addict and addicted mice using PCA analysis. Top varying 500 genes were selected for PCA analysis. “nbinomTest” function used to calculate p-value from DEseq package. Only those genes were considered as differentially expressed genes that fulfil the criteria of at least a 1.5 fold change, a p-value less than 0.05, a FDR less than 0.1 and at least 10 read counts in either condition from both cases of addiction. Volcano plots, boxplots were plotted using ggplot2 package in R.

### Gene ontology and protein–protein interaction network analysis

Gene ontology analysis for differentially expressed shared genes were performed using ToppGene^51^. Protein–protein interactions were predicted using STRING database^15^. In this analysis, experimental data, co-expressed genes, neighboring genes, other databases and text mining from literature were used for predicting the PPI network. Here we used the default parameter of the confidence score (0.4) provided by database to generate most likely interactions. We implemented Cytoscape^52^ (version 3.8.0) to visualize the network by importing protein–protein interaction predicted by STRING database.

### Single cell RNA-sequencing analysis

Single cell expression matrix was obtained from the NCBI-GEO portal. Matrix was curate for the cells with cocaine self-administration mice during the maintenance phase condition. Selected matrix was processed with seurat^53^ in R. Gene variability were calculated using function “FindVariableFeatures”. Using “RunPCA” function principle components were calculated. Further cluster analysis was performed using Seurat-inbuilt function “FindClusters”. Further clusters were visualized using the “RunTSNE” function. Clusters were annotated using the expression of the known marker genes (Supplement Fig. 1). Heatmaps were plotted using package “pheatmap” in R.

## Supplementary Information

Supplementary Table S1.

Supplementary Table S2.

Supplementary Table S3.

Supplementary Figure 1.

Supplementary Figure 2.

Supplementary Figure 3.

Supplementary Figure 4.
