# Supplementary material for: Transcriptional signatures in prefrontal cortex confer vulnerability versus resilience to food and cocaine addiction-like behavior
Source: Sci Rep. 2021 Apr 27;11:9076. doi: 10.1038/s41598-021-88363-9 (PMC8079697; doi:10.1038/s41598-021-88363-9)
Supplement: Supplementary file 1 — Supplementary Table S1. [file 41598_2021_88363_MOESM1_ESM.pdf]

Transcriptional signatures in prefrontal cortex confer vulnerability versus resilience to food and cocaine addiction-like behavior

Mohit Navandar[1], Elena Martín-García[2], Rafael Maldonado[2,3], Beat Lutz[4,5], Susanne Gerber[1#], Inigo Ruiz de Azua[4,5#]

[1] Institute for Human Genetics, University Medical Center of the Johannes Gutenberg University Mainz, Mainz, Germany

[2] Laboratory of Neuropharmacology-Neurophar, Department of Experimental and Health Sciences, Universitat Pompeu Fabra (UPF), Barcelona, Spain.

[3] Hospital del Mar Medical Research Institute (IMIM), Barcelona, Spain.

[4] Institute of Physiological Chemistry, University Medical Center of the Johannes Gutenberg University Mainz, Mainz, Germany

[5] Leibniz Institute for Resilience Research (LIR), Mainz, Germany

[#] These authors jointly supervised this work

Corresponding author: Inigo Ruiz de Azua. E-mail: Inigo.azua@lir-mainz.de

Supplementary table S1: List of unique differentially expressed genes in palatable food addicted vs non-addicted mice

Genes	Non-addiction (Mean Reads)	Addicted (Mean Reads)	log2 Fold Change	P-value	padj	Type
Mid1	110.7075	169.3694	0.61342	8.95E-08	1.57E-05	Upregulated
Rph3al	19.74357	30.38982	0.622206	0.001374	0.06367	Upregulated
Hspa1a	163.1025	271.3451	0.73435	1.63E-14	1.14E-11	Upregulated
Hspa1b	203.4112	349.4882	0.780845	1.25E-17	1.08E-14	Upregulated
Hist1h3c	12.5512	22.67916	0.853541	0.000226	0.01487	Upregulated
Gpr139	9.930581	18.42438	0.891666	0.000386	0.022814	Upregulated
Arhgap36	8.672425	16.41459	0.920471	0.000755	0.039567	Upregulated
Hist1h3g	11.5166	22.38788	0.959003	4.7E-05	0.003934	Upregulated
Capn11	9.749046	19.60873	1.008163	6.86E-05	0.00544	Upregulated
Dgkk	5.511845	11.34607	1.041586	0.001571	0.071105	Upregulated
Hist1h2af	6.332554	13.1409	1.053205	0.000609	0.033422	Upregulated
Slc18a3	7.304863	17.82558	1.28702	1.64E-06	0.000211	Upregulated
Lhx8	3.805638	15.34677	2.011725	3.34E-10	1.11E-07	Upregulated
Cyp2f2	2.418482	10.34864	2.097267	2.14E-07	3.36E-05	Upregulated
Emilin1	93.11205	62.143	-0.58338	3.76E-06	0.000439	Downregulated
Grem1	35.39033	23.59363	-0.58496	0.001031	0.051057	Downregulated
Tspan11	41.87934	27.89118	-0.58643	0.000475	0.027114	Downregulated
Cyp26b1	130.0908	86.56031	-0.58774	1.02E-07	1.78E-05	Downregulated
Nupr1	80.02454	53.11965	-0.5912	5.76E-06	0.000644	Downregulated
Vipr2	183.3122	121.2103	-0.59679	1.56E-08	3.34E-06	Downregulated
Mrc1	189.9958	125.5506	-0.5977	4.63E-09	1.18E-06	Downregulated
Ctxn3	48.30369	31.91897	-0.59772	0.000284	0.017847	Downregulated
Shisa3	178.831	117.9156	-0.60084	6.72E-09	1.56E-06	Downregulated
Hpse	44.43219	29.27935	-0.60172	0.000279	0.017719	Downregulated
Frmd7	624.8698	411.6567	-0.60211	4.4E-13	2.37E-10	Downregulated
Mgp	275.7015	181.6125	-0.60224	1.06E-10	3.82E-08	Downregulated
Acta2	452.4503	297.5415	-0.60467	1.91E-12	9.35E-10	Downregulated
Fibin	71.39925	46.88618	-0.60675	1.33E-05	0.001369	Downregulated
Fosb	340.582	223.0157	-0.61086	6.3E-10	1.97E-07	Downregulated
Postn	68.0255	44.54222	-0.6109	1.39E-05	0.001416	Downregulated
Aqp1	119.8856	77.87374	-0.62245	2.96E-08	5.8E-06	Downregulated
Adamts19	297.277	192.7193	-0.62531	1.76E-11	7.29E-09	Downregulated
Clec1a	38.28189	24.81661	-0.62536	0.000692	0.036596	Downregulated
Myh11	576.2763	372.226	-0.63058	4.01E-14	2.62E-11	Downregulated
Plscr2	38.91649	25.13404	-0.63074	0.000288	0.01792	Downregulated
Klhl6	39.64621	25.58414	-0.63193	0.000376	0.022423	Downregulated
Sfrp4	28.30082	18.22075	-0.63526	0.001244	0.058945	Downregulated
Adm	33.83672	21.78106	-0.63552	0.001144	0.055889	Downregulated
F13a1	94.48727	60.76323	-0.63692	4.31E-07	6.2E-05	Downregulated
Osr1	46.57378	29.8102	-0.64371	3.29E-05	0.002973	Downregulated

Slc6a13	380.1194	242.8111	-0.64662	1.28E-13	7.68E-11	Downregulated
Mrc2	314.9115	200.8114	-0.64911	1.11E-12	5.63E-10	Downregulated
Lrrc32	85.0196	54.1089	-0.65193	1.47E-06	0.00019	Downregulated
Msln	28.95218	18.4117	-0.65305	0.001044	0.051574	Downregulated
Cnn1	37.20101	23.58977	-0.65718	0.000301	0.018545	Downregulated
Anxa1	33.87133	21.47102	-0.65767	0.000366	0.021913	Downregulated
Tfap2b	21.02722	13.30432	-0.66036	0.002095	0.088153	Downregulated
Trh	180.8	113.2157	-0.67532	1.56E-10	5.35E-08	Downregulated
Npffr2	53.16797	33.22963	-0.67809	2.07E-05	0.002006	Downregulated
Col13a1	49.82812	30.98051	-0.6856	1.47E-05	0.001486	Downregulated
Bmp5	30.04901	18.6797	-0.68585	0.0004	0.023549	Downregulated
Gpnmb	125.3062	77.44532	-0.69421	1.61E-09	4.64E-07	Downregulated
Shisa8	335.4081	206.8998	-0.69699	1.98E-13	1.12E-10	Downregulated
Svep1	128.659	79.28489	-0.69844	7.11E-10	2.19E-07	Downregulated
Akr1c14	23.70156	14.59361	-0.69965	0.001644	0.073738	Downregulated
Espnl	30.48573	18.7465	-0.70151	0.000282	0.017804	Downregulated
Igf2	757.0006	461.1675	-0.715	1.64E-18	1.61E-15	Downregulated
Gdpd4	21.98947	13.31918	-0.72331	0.000928	0.0466	Downregulated
Siglec1	36.22638	21.91005	-0.72545	5.64E-05	0.004648	Downregulated
Ptgdr	31.52475	19.05147	-0.72658	9.29E-05	0.006993	Downregulated
8430408G22Rik	41.18932	24.74797	-0.73496	6.25E-05	0.005056	Downregulated
Thbs1	127.2323	76.07601	-0.74195	3.35E-10	1.11E-07	Downregulated
Olfr115	23.30523	13.90105	-0.74546	0.001245	0.058945	Downregulated
Slc22a2	29.36193	17.4894	-0.74747	4.36E-05	0.003707	Downregulated
Ogn	216.404	128.8283	-0.74828	4.99E-15	3.85E-12	Downregulated
Igfbp2	723.1414	430.3394	-0.7488	5.45E-19	5.6E-16	Downregulated
Cytl1	21.37443	12.71842	-0.74897	0.000917	0.046371	Downregulated
Ptgis	70.57042	41.96467	-0.74989	6.5E-08	1.18E-05	Downregulated
Sphk1	94.21715	55.80844	-0.75551	1.82E-09	5.09E-07	Downregulated
Crispld2	73.28988	43.39205	-0.75618	5.27E-08	9.72E-06	Downregulated
Prg4	49.2393	29.1434	-0.75664	4.31E-06	0.0005	Downregulated
Ppm1j	71.79726	42.46684	-0.75759	8.15E-08	1.46E-05	Downregulated
Slc6a20a	341.0822	201.5842	-0.75874	8.88E-17	7.37E-14	Downregulated
Gjb2	210.8535	124.3091	-0.76231	3.04E-14	2.05E-11	Downregulated
C1qtnf7	19.60159	11.41101	-0.78054	0.001255	0.059009	Downregulated
S100a5	54.44701	31.65568	-0.78239	2.51E-07	3.87E-05	Downregulated
Trdn	43.31477	24.68371	-0.8113	2.57E-06	0.000311	Downregulated
Bdkrb2	17.93348	10.19713	-0.81449	0.001911	0.082341	Downregulated
Olfr78	26.71061	15.17423	-0.81579	6.43E-05	0.00514	Downregulated
Fgl2	94.23829	53.1606	-0.82596	1.01E-10	3.69E-08	Downregulated
Foxd1	47.40528	26.73722	-0.8262	3.19E-07	4.79E-05	Downregulated
Hcar1	26.74573	14.99916	-0.83443	4.79E-05	0.003979	Downregulated
Mpzl2	48.36085	27.11437	-0.83478	1.68E-07	2.72E-05	Downregulated
Slc6a12	46.43178	25.91339	-0.84141	3.46E-07	5.12E-05	Downregulated
Prdm6	16.00573	8.895932	-0.84737	0.000988	0.049162	Downregulated
Fmod	342.9951	189.2918	-0.85758	2.06E-21	2.61E-18	Downregulated

Cyp1b1	128.38	70.54726	-0.86376	1.57E-13	9.17E-11	Downregulated
Aldh1a2	232.667	126.0162	-0.88466	4.61E-19	4.98E-16	Downregulated
Ptgds	10011.7	5410.763	-0.88778	2.72E-34	1.47E-30	Downregulated
Wnt6	65.68514	35.32181	-0.89501	3.93E-10	1.26E-07	Downregulated
Slc26a7	39.19592	20.96774	-0.90253	3.21E-07	4.79E-05	Downregulated
Fat2	19.19549	10.2251	-0.90865	0.000213	0.014173	Downregulated
Crabp2	50.73144	27.01937	-0.90889	2.21E-08	4.54E-06	Downregulated
H2-Ab1	49.11281	26.13347	-0.9102	2.85E-08	5.65E-06	Downregulated
Foxc2	37.82868	20.10538	-0.9119	5.65E-07	7.9E-05	Downregulated
Cdh1	48.69094	25.85654	-0.91312	9.11E-09	2.05E-06	Downregulated
Lrg1	25.63233	13.57165	-0.91737	3.7E-05	0.003257	Downregulated
Pf4	14.07126	7.439515	-0.91947	0.001774	0.077897	Downregulated
Cd74	122.1143	64.54366	-0.91989	5.78E-15	4.3E-12	Downregulated
Mrgprf	26.91744	14.16415	-0.9263	9.41E-06	0.001001	Downregulated
Cd209f	15.2193	7.980917	-0.93128	0.001056	0.052061	Downregulated
Ifitm1	36.09152	18.34093	-0.97659	2.66E-07	4.08E-05	Downregulated
Hephl1	39.20127	19.25691	-1.02552	3.04E-08	5.91E-06	Downregulated
Slc22a6	242.0157	117.7981	-1.03878	4.62E-26	1.11E-22	Downregulated
H2-Aa	57.47972	27.7664	-1.04971	1.49E-11	6.57E-09	Downregulated
Maff	55.44815	25.93006	-1.09651	9.39E-11	3.51E-08	Downregulated
Il13ra2	13.87627	6.348502	-1.12813	0.000133	0.009666	Downregulated
H2-Q1	16.98185	7.715273	-1.1382	1.65E-05	0.001636	Downregulated
Slc45a2	10.97326	4.984039	-1.13861	0.000987	0.049162	Downregulated
Tyrp1	45.84567	20.33049	-1.17314	1.64E-11	6.96E-09	Downregulated
Il31ra	10.48306	4.125046	-1.34558	0.000242	0.015687	Downregulated
Avp	19.70503	5.681145	-1.79431	7.65E-10	2.32E-07	Downregulated
