# Supplementary material for: Transcriptional signatures in prefrontal cortex confer vulnerability versus resilience to food and cocaine addiction-like behavior
Source: Sci Rep. 2021 Apr 27;11:9076. doi: 10.1038/s41598-021-88363-9 (PMC8079697; doi:10.1038/s41598-021-88363-9)
Supplement: Supplementary file 2 — Supplementary Table S2. [file 41598_2021_88363_MOESM2_ESM.pdf]

Transcriptional signatures in prefrontal cortex confer vulnerability versus resilience to food and cocaine addiction-like behavior

Mohit Navandar[1], Elena Martín-García[2], Rafael Maldonado[2,3], Beat Lutz[4,5], Susanne Gerber[1#], Inigo Ruiz de Azua[4,5#]

[1] Institute for Human Genetics, University Medical Center of the Johannes Gutenberg University Mainz, Mainz, Germany

[2] Laboratory of Neuropharmacology-Neurophar, Department of Experimental and Health Sciences, Universitat Pompeu Fabra (UPF), Barcelona, Spain.

[3] Hospital del Mar Medical Research Institute (IMIM), Barcelona, Spain.

[4] Institute of Physiological Chemistry, University Medical Center of the Johannes Gutenberg University Mainz, Mainz, Germany

[5] Leibniz Institute for Resilience Research (LIR), Mainz, Germany

[#] These authors jointly supervised this work

Corresponding author: Inigo Ruiz de Azua. E-mail: Inigo.azua@lir-mainz.de

Supplementary table S2: List of unique differentially expressed genes in cocaine addicted vs non-addicted mice

Genes	Non-addiction (Mean Reads)	Addicted (Mean Reads)	log2 Fold Change	P-value	padj	Type
Gatm	2108.036	3153.181	0.580908	1.17E-08	1.4E-06	Upregulated
Prr18	931.9925	1394.787	0.581655	6.21E-08	6.68E-06	Upregulated
Gm19522	34.1375	51.10573	0.582128	0.003383	0.082295	Upregulated
Mdga1	296.0948	443.4377	0.582672	7.99E-08	8.42E-06	Upregulated
Mbp	43993.34	65935.52	0.583771	8.96E-09	1.13E-06	Upregulated
Gjc3	1110.064	1665.746	0.585525	4.68E-08	5.15E-06	Upregulated
Mcm3	59.69481	89.68464	0.587256	0.000269	0.010179	Upregulated
Serping1	96.49081	145.0577	0.588163	2.46E-05	0.001304	Upregulated
Synpo2	109.8997	165.2371	0.58835	1.97E-05	0.001087	Upregulated
Cdkn2c	36.04007	54.23215	0.589546	0.001887	0.051483	Upregulated
Cfap70	40.66767	61.20273	0.589714	0.000997	0.030543	Upregulated
Dlx6os1	405.7338	611.6628	0.592203	5.11E-08	5.56E-06	Upregulated
Prr5l	214.833	324.1439	0.593419	1.55E-06	0.000117	Upregulated
Trim59	295.3366	446.2748	0.595572	2.85E-07	2.73E-05	Upregulated
Galnt6	319.5396	482.8821	0.595676	2.51E-07	2.45E-05	Upregulated
Egr2	531.6594	804.1231	0.596914	6.97E-09	9.14E-07	Upregulated
Fam83d	60.67572	91.7797	0.597056	0.000179	0.007158	Upregulated
Lrrc9	54.20858	82.09877	0.598839	0.000242	0.009348	Upregulated
Dlx6	66.37382	100.5281	0.598913	0.000159	0.006556	Upregulated
Efemp1	116.9803	177.2488	0.59951	4.57E-06	0.000299	Upregulated
Lrrc1	190.3854	288.7556	0.600926	8.06E-07	6.69E-05	Upregulated
Nmbr	31.92571	48.43024	0.60119	0.001827	0.050337	Upregulated
Itga10	103.8198	157.5024	0.601292	1.85E-06	0.000135	Upregulated
Vstm4	51.35854	77.98059	0.602511	0.00044	0.015435	Upregulated
Kif13b	587.2644	891.9456	0.602945	2.32E-08	2.63E-06	Upregulated
Mag	3072.642	4668.305	0.603419	5.55E-09	7.38E-07	Upregulated
Dach1	176.9668	269.3222	0.605854	1.4E-06	0.000107	Upregulated
A230001M10Rik	40.15464	61.11519	0.605964	0.001947	0.052443	Upregulated
Slain1os	27.28126	41.53192	0.60631	0.003708	0.088201	Upregulated
Chdh	129.6231	197.4709	0.607318	2.78E-06	0.000191	Upregulated
Strip2	538.9684	822.7065	0.610177	1.31E-08	1.53E-06	Upregulated
Gpr37	1136.853	1735.811	0.610564	1.25E-08	1.49E-06	Upregulated
C030029H02Rik	67.91617	103.7774	0.611666	0.00011	0.004759	Upregulated
Ndnf	259.6329	396.7768	0.611854	7.62E-08	8.07E-06	Upregulated
Fam183b	31.83211	48.71309	0.613826	0.001457	0.042449	Upregulated
Mobp	5195.236	7950.442	0.613846	2.26E-09	3.25E-07	Upregulated
Il16	117.5186	179.9723	0.614885	1.59E-06	0.000119	Upregulated
Tmem88b	1140.985	1748.14	0.61554	8.42E-09	1.07E-06	Upregulated
Tmem63a	651.0716	999.6718	0.618638	1.04E-08	1.28E-06	Upregulated
Kcna5	114.0272	175.4396	0.621597	4.46E-06	0.000292	Upregulated
B230206H07Rik	32.80859	50.5116	0.622541	0.002	0.053302	Upregulated

Dlx2	139.1404	214.8362	0.626696	1.37E-06	0.000105	Upregulated
Syne3	41.96006	64.81972	0.627416	0.000456	0.015796	Upregulated
Plk5	164.6607	254.5877	0.628666	3.36E-07	3.14E-05	Upregulated
Cldn11	2866.844	4433.866	0.629102	1.16E-09	1.75E-07	Upregulated
Fam167a	72.172	111.7556	0.630836	2.15E-05	0.001171	Upregulated
Dnah9	123.0421	191.0428	0.634744	7.76E-07	6.47E-05	Upregulated
Cpm	284.0369	441.0726	0.634937	2.88E-08	3.2E-06	Upregulated
Fa2h	697.5217	1091.19	0.645593	2.75E-09	3.83E-07	Upregulated
Plekhf1	110.8768	173.5481	0.646379	1.67E-06	0.000125	Upregulated
Trf	3808.174	5965.146	0.647458	5.01E-10	8.16E-08	Upregulated
Adamts4	394.3724	618.1698	0.648445	1.51E-08	1.74E-06	Upregulated
Il33	756.8479	1188.237	0.650747	1.42E-09	2.11E-07	Upregulated
Mmp23	22.39548	35.21623	0.653033	0.002024	0.053754	Upregulated
Myrf	1238.997	1948.582	0.653252	4.58E-10	7.57E-08	Upregulated
Slc25a13	37.2557	58.62897	0.654153	0.000351	0.012742	Upregulated
Pcp4l1	1035.779	1634.438	0.658079	5.78E-10	9.25E-08	Upregulated
Clec3b	24.69749	39.1305	0.663929	0.002193	0.057186	Upregulated
Plp1	29612.49	46922.18	0.664064	6.1E-11	1.18E-08	Upregulated
Mme	137.3166	217.6895	0.664766	2.86E-07	2.73E-05	Upregulated
Enpp1	100.6718	159.8001	0.666608	2.61E-06	0.000183	Upregulated
Cdca8	26.7644	42.5128	0.667582	0.001883	0.051483	Upregulated
Enpp6	107.3643	170.9542	0.671096	1.14E-06	9.05E-05	Upregulated
Actn2	247.5452	394.4465	0.672138	1.01E-08	1.25E-06	Upregulated
Ccna2	52.87316	84.46912	0.675888	4.49E-05	0.002196	Upregulated
Plekhh1	709.1221	1134.938	0.678508	2.77E-10	4.73E-08	Upregulated
Tnfaip6	181.1924	290.0482	0.678771	5.96E-08	6.45E-06	Upregulated
Gabrr2	25.5048	40.85153	0.679621	0.001215	0.036183	Upregulated
Evi2a	276.2252	442.9018	0.681142	4.8E-09	6.46E-07	Upregulated
Sox2ot	525.0354	842.566	0.682375	5.57E-10	8.98E-08	Upregulated
Foxm1	46.55297	74.75515	0.6833	5.42E-05	0.002597	Upregulated
Dnaaf3	23.12634	37.15636	0.684072	0.001716	0.047749	Upregulated
Ninj2	25.41499	40.8669	0.685253	0.002047	0.054288	Upregulated
Fbxw15	25.78919	41.49838	0.686289	0.001342	0.039377	Upregulated
Stk36	100.535	161.7798	0.686334	1.76E-07	1.79E-05	Upregulated
Cenpe	26.7024	43.12173	0.691446	0.000987	0.03037	Upregulated
Necab2	867.4077	1404.405	0.695176	1.54E-11	3.23E-09	Upregulated
Dock10	779.5613	1262.887	0.695991	5.56E-11	1.09E-08	Upregulated
Bcas1	2022.042	3282.977	0.699191	1.25E-11	2.7E-09	Upregulated
Spag8	35.94038	58.43057	0.701118	0.000142	0.006003	Upregulated
Kif6	37.64016	61.20843	0.701458	0.000158	0.006542	Upregulated
Espl1	28.71157	46.7877	0.704497	0.000488	0.016638	Upregulated
Shisa2	92.97427	151.5388	0.704784	8.63E-07	7.03E-05	Upregulated
Zfp503	43.11317	70.34254	0.706269	8.32E-05	0.003819	Upregulated
Itgb4	202.288	330.22	0.707017	7.88E-09	1.01E-06	Upregulated
Gng7	2912.466	4767.141	0.710883	3.49E-12	8.16E-10	Upregulated
Grid2ip	51.84886	84.86886	0.710923	4.21E-06	0.000277	Upregulated

Cyp39a1	28.70614	47.01396	0.71173	0.000518	0.01757	Upregulated
Rasd2	1266.828	2076.254	0.712762	8.38E-12	1.87E-09	Upregulated
Aox3	33.4991	54.95199	0.714049	0.000102	0.004466	Upregulated
Chia1	31.38674	51.5562	0.715991	0.000216	0.008527	Upregulated
Sox1	286.5102	471.7041	0.719295	1.68E-10	3.02E-08	Upregulated
Slc12a8	22.46	37.04949	0.722096	0.00071	0.022931	Upregulated
Hapln2	355.1229	587.1867	0.725501	1.24E-10	2.3E-08	Upregulated
Gfap	2241.754	3707.496	0.725817	5.85E-13	1.47E-10	Upregulated
Mamdc2	23.24443	38.44369	0.725862	0.000993	0.030506	Upregulated
Maats1	43.42626	71.90464	0.727517	2.01E-05	0.001104	Upregulated
P2rx5	40.62404	67.27232	0.727679	1.23E-05	0.000714	Upregulated
Hhip	210.8151	350.1506	0.731997	1.1E-09	1.68E-07	Upregulated
Dlec1	51.64699	85.82925	0.732785	4.04E-06	0.000267	Upregulated
Insc	71.88733	119.6537	0.735056	8.39E-07	6.94E-05	Upregulated
Myh6	69.95751	116.6943	0.738183	4.11E-07	3.79E-05	Upregulated
Ano1	76.2945	127.3918	0.739622	4.49E-07	4.04E-05	Upregulated
Kntc1	18.59089	31.1009	0.74236	0.001508	0.043617	Upregulated
Rxrg	204.3311	341.8464	0.742439	1.11E-09	1.68E-07	Upregulated
Erbb3	188.3821	315.209	0.742647	7.97E-10	1.24E-07	Upregulated
Ugt8a	1017.968	1712.582	0.750481	2.08E-12	4.97E-10	Upregulated
Prkch	101.34	170.5181	0.75072	6.75E-08	7.18E-06	Upregulated
Armc3	17.08124	28.77987	0.752647	0.00397	0.093014	Upregulated
1110017D15Rik	24.51461	41.32718	0.753449	0.000176	0.007097	Upregulated
Gpr149	17.40647	29.3752	0.754975	0.001958	0.05269	Upregulated
Fzd5	49.48129	83.5065	0.755005	4.83E-06	0.000313	Upregulated
Mst1	13.3287	22.57491	0.760184	0.002415	0.062331	Upregulated
Abca8a	188.4444	319.3195	0.760862	2.69E-10	4.67E-08	Upregulated
Cd24a	187.0283	316.9396	0.760951	2.14E-10	3.76E-08	Upregulated
Gjb1	163.7642	277.9703	0.763311	1.34E-09	2.01E-07	Upregulated
Slc22a3	19.00639	32.295	0.764826	0.001078	0.032781	Upregulated
C4b	435.5703	740.2847	0.765175	1.52E-12	3.69E-10	Upregulated
Tpx2	51.21594	87.10691	0.766194	2.5E-06	0.000176	Upregulated
2410004P03Rik	48.01327	81.81461	0.768925	7.68E-06	0.000478	Upregulated
Hist1h2be	41.16029	70.31008	0.772479	1.5E-05	0.000848	Upregulated
Bmp6	65.48009	112.1938	0.776865	4.69E-07	4.17E-05	Upregulated
Scnn1a	39.28629	67.423	0.779215	1.36E-05	0.000785	Upregulated
St8sia6	22.36113	38.4203	0.780876	0.000341	0.012416	Upregulated
Col6a5	17.09855	29.38971	0.781437	0.001619	0.045615	Upregulated
Mboat1	52.67372	90.54207	0.781505	1.28E-06	9.95E-05	Upregulated
Tex15	19.66842	33.84891	0.783228	0.000383	0.013671	Upregulated
Sp9	104.6309	180.2732	0.784876	7.62E-09	9.88E-07	Upregulated
Stk32a	59.42447	102.5876	0.787727	4.58E-07	4.1E-05	Upregulated
Pde1c	199.7352	344.9652	0.788362	4.86E-11	9.6E-09	Upregulated
Knstrn	32.88084	56.89971	0.791174	1.66E-05	0.000935	Upregulated
Rorc	27.03047	46.8308	0.792871	9.75E-05	0.004328	Upregulated
Cd163	40.59441	70.46179	0.79556	6.11E-06	0.000386	Upregulated

Cryab	1446.294	2515.318	0.79838	8.07E-15	2.83E-12	Upregulated
Ttc16	18.8507	32.79253	0.798749	0.000491	0.016731	Upregulated
Sspo	75.42271	131.3345	0.800175	2.47E-08	2.78E-06	Upregulated
Plin4	290.4867	505.9797	0.800607	3.34E-13	8.6E-11	Upregulated
Arhgap6	118.9192	207.522	0.803282	5.94E-10	9.43E-08	Upregulated
Ubxn10	32.96128	57.55866	0.804261	2.54E-05	0.001342	Upregulated
Adamtsl4	105.7694	184.8909	0.805753	1.85E-09	2.71E-07	Upregulated
Klk6	16.80214	29.39953	0.807148	0.001636	0.046037	Upregulated
A330049N07Rik	17.05988	29.8513	0.807187	0.001328	0.039155	Upregulated
A230009B12Rik	23.5904	41.28945	0.807574	0.000167	0.006854	Upregulated
Gm13293	25.76629	45.12954	0.808587	0.000102	0.004466	Upregulated
Smco3	133.1201	233.1881	0.808766	3.33E-10	5.6E-08	Upregulated
Zic5	28.42626	49.84036	0.81009	3.45E-05	0.001749	Upregulated
Ccdc162	14.11622	24.758	0.81054	0.003103	0.076679	Upregulated
Plekhg4	49.67866	87.16126	0.811061	3.06E-07	2.91E-05	Upregulated
Cfap43	59.59229	104.5639	0.811187	2.02E-07	2.04E-05	Upregulated
Cyp2j12	51.30938	90.16594	0.81336	9.9E-07	7.96E-05	Upregulated
Tekt1	25.39492	44.63166	0.813528	0.000123	0.005265	Upregulated
Fam196b	30.67635	53.9576	0.814699	2.43E-05	0.001294	Upregulated
Plxnb3	571.7925	1006.346	0.815562	2E-14	6.56E-12	Upregulated
Ccdc40	45.49384	80.08985	0.815948	8.48E-07	6.96E-05	Upregulated
Stpg1	12.66642	22.31956	0.817299	0.003357	0.08178	Upregulated
Prima1	35.52185	62.95685	0.825657	1.13E-05	0.000666	Upregulated
Kif19a	55.88938	99.11366	0.82651	2.2E-07	2.21E-05	Upregulated
Cyr61	246.2617	436.9666	0.827331	2.01E-13	5.52E-11	Upregulated
Pipox	21.11807	37.53116	0.829611	0.000259	0.009874	Upregulated
Agbl2	21.20143	37.93782	0.839475	0.000176	0.007101	Upregulated
Zbtb20	56.05331	100.3954	0.840822	9.3E-08	9.7E-06	Upregulated
Zic2	241.9553	433.9665	0.842843	2.34E-13	6.31E-11	Upregulated
Npas4	970.8371	1743.565	0.844739	3.41E-17	1.61E-14	Upregulated
Prr11	13.4467	24.15611	0.845136	0.001375	0.040231	Upregulated
Rrm2	41.0136	73.82873	0.84808	1.31E-06	0.000102	Upregulated
Rasgrp2	292.0062	525.9426	0.848906	1.05E-13	3.04E-11	Upregulated
Nuf2	34.6188	62.46378	0.851464	3.03E-06	0.000206	Upregulated
Sncaip	60.29501	108.8389	0.852084	2.98E-08	3.29E-06	Upregulated
Mxd3	15.06512	27.20423	0.852619	0.000848	0.026623	Upregulated
Gm11992	16.2354	29.42279	0.857791	0.000565	0.018946	Upregulated
Ccdc81	19.73454	35.76958	0.85801	0.000148	0.006207	Upregulated
Dnaic2	30.79872	55.91378	0.860334	1.12E-05	0.000659	Upregulated
Map3k15	55.6985	101.1434	0.860692	1.1E-08	1.35E-06	Upregulated
Gm5607	190.8838	346.983	0.86217	2.86E-13	7.49E-11	Upregulated
Cacng5	242.1141	441.0008	0.865094	1.46E-14	4.86E-12	Upregulated
Ermn	1178.261	2146.357	0.865231	2.81E-16	1.22E-13	Upregulated
Sec14l5	181.7418	331.0839	0.865306	6.38E-13	1.58E-10	Upregulated
Adgb	17.6637	32.26735	0.869288	0.000282	0.010612	Upregulated
Spc25	13.02423	23.82661	0.871376	0.000997	0.030543	Upregulated

Myo3b	20.12717	36.85483	0.87271	0.000121	0.005197	Upregulated
D7Ertd443e	62.65076	114.8275	0.874064	1.1E-08	1.35E-06	Upregulated
Iqca	27.32728	50.11969	0.875035	2.34E-05	0.001254	Upregulated
A2m	40.72138	74.68676	0.875066	3.97E-07	3.69E-05	Upregulated
Pbx3	133.2401	245.022	0.878883	1.46E-11	3.09E-09	Upregulated
Serpind1	21.30882	39.19541	0.879234	7.77E-05	0.003601	Upregulated
Rem2	124.1167	229.1503	0.884596	9.95E-12	2.18E-09	Upregulated
Mki67	107.0234	199.0861	0.895466	1.78E-11	3.7E-09	Upregulated
Rsph1	35.55806	66.24773	0.897694	7.47E-07	6.28E-05	Upregulated
Serpinb1a	76.48693	142.5555	0.898238	4.8E-10	7.87E-08	Upregulated
Col8a2	29.57971	55.15399	0.898857	3.76E-06	0.000252	Upregulated
Mir212	13.32141	24.8632	0.900266	0.000537	0.01814	Upregulated
Bbox1	19.36696	36.23259	0.903691	9.45E-05	0.004243	Upregulated
Cdca7l	10.68218	20.00633	0.905251	0.00173	0.048079	Upregulated
Pde7b	349.6264	655.262	0.906258	1.19E-15	4.49E-13	Upregulated
Dock5	252.062	472.4962	0.906524	3.23E-15	1.2E-12	Upregulated
Fbln7	34.56795	64.92996	0.909449	8.45E-07	6.95E-05	Upregulated
Cfap57	22.62081	42.69857	0.916537	1.94E-05	0.001072	Upregulated
Arhgef39	9.505098	17.96582	0.918482	0.003172	0.077849	Upregulated
Rasd1	141.9906	268.5346	0.919312	3.51E-14	1.11E-11	Upregulated
Lbp	40.17084	76.09349	0.921624	2.81E-07	2.71E-05	Upregulated
Ripk4	11.20401	21.2511	0.923522	0.001565	0.044634	Upregulated
Fam179a	46.63792	89.07025	0.93344	2.74E-08	3.06E-06	Upregulated
Dynlrb2	13.28008	25.43318	0.937448	0.000364	0.01308	Upregulated
Sv2c	125.6156	241.0876	0.940543	8.41E-14	2.48E-11	Upregulated
1700007K13Rik	35.69973	68.76136	0.945685	2.63E-07	2.55E-05	Upregulated
Prokr2	52.10073	100.5012	0.947837	1.7E-09	2.51E-07	Upregulated
Krt2	9.212046	17.79186	0.949624	0.001649	0.046331	Upregulated
St18	140.3533	271.1892	0.950237	5.01E-14	1.54E-11	Upregulated
Mns1	24.43162	47.21645	0.95054	5.93E-06	0.000377	Upregulated
Ccdc114	42.66003	82.51889	0.951839	9.65E-09	1.2E-06	Upregulated
Anln	710.9103	1381.288	0.958275	3.26E-19	1.84E-16	Upregulated
Bdh2	9.556518	18.59767	0.960565	0.001583	0.04502	Upregulated
Ntf3	25.99215	50.59498	0.960918	1.35E-06	0.000104	Upregulated
9330117O12Rik	22.58008	44.16962	0.968004	9.08E-06	0.000553	Upregulated
Dnah10	49.81627	97.76946	0.972767	6.36E-10	1E-07	Upregulated
Pou3f4	41.2861	82.12142	0.992103	5.63E-09	7.44E-07	Upregulated
Ttr	18.70694	37.31433	0.996156	3.92E-06	0.000261	Upregulated
Ankfn1	14.39156	28.74182	0.997928	7.27E-05	0.003399	Upregulated
Prrg4	10.95862	21.951	1.002221	0.000434	0.015303	Upregulated
Mob3b	90.14387	182.7092	1.019248	6.22E-14	1.89E-11	Upregulated
Ect2	18.58443	37.85824	1.026513	6.44E-06	0.000405	Upregulated
Ckap2l	20.32672	41.41318	1.026713	2.74E-06	0.000189	Upregulated
Sstr5	9.609996	19.63507	1.030825	0.000799	0.025402	Upregulated
Aspm	22.59032	46.16229	1.03101	1.32E-06	0.000102	Upregulated
Kif11	41.07398	84.28212	1.037002	7.73E-10	1.21E-07	Upregulated

Ppp1r32	15.37776	31.69838	1.043564	2.45E-05	0.0013	Upregulated
Ccdc33	9.87027	20.4131	1.048334	0.00034	0.012398	Upregulated
Casc1	14.80287	30.62343	1.048759	3.02E-05	0.001562	Upregulated
Uhrf1	22.32109	46.21284	1.049886	4.61E-07	4.11E-05	Upregulated
Lrrc71	17.13059	36.06059	1.073848	5.22E-06	0.000334	Upregulated
Fam64a	7.743755	16.33346	1.076725	0.00121	0.036098	Upregulated
Rarb	178.3104	377.159	1.080782	3.93E-19	2.1E-16	Upregulated
Armc4	10.22882	21.68276	1.083908	0.000222	0.008714	Upregulated
Lect1	8.87463	18.86516	1.087965	0.000482	0.016493	Upregulated
Ankrd66	5.551835	11.83854	1.092454	0.00435	0.099749	Upregulated
Tcp11	11.10672	23.72012	1.094678	6.53E-05	0.003068	Upregulated
Cdca7	57.77985	124.4105	1.10647	1.85E-13	5.15E-11	Upregulated
Ticrr	5.552405	11.96603	1.10776	0.003759	0.089235	Upregulated
Drc7	30.49883	65.78516	1.109008	4.04E-09	5.47E-07	Upregulated
Zic1	360.8721	779.0981	1.110317	9.82E-25	7.68E-22	Upregulated
Gm867	9.815688	21.39169	1.123889	0.000159	0.006565	Upregulated
Fam166b	10.02763	21.93273	1.129104	0.000163	0.006712	Upregulated
4933413G19Rik	14.49148	31.90955	1.138784	3.4E-06	0.000229	Upregulated
Ccdc24	14.58992	32.17245	1.140854	2.95E-06	0.000202	Upregulated
Lrrc23	32.18687	70.99427	1.14123	1.55E-10	2.81E-08	Upregulated
Sp8	60.31823	133.355	1.144606	6.29E-15	2.25E-12	Upregulated
Ckap2	12.06815	26.73427	1.147486	1.79E-05	0.000993	Upregulated
Cfap52	16.94978	37.75691	1.155475	4.22E-07	3.85E-05	Upregulated
Bfsp2	70.98934	158.4267	1.158142	2.88E-16	1.22E-13	Upregulated
Draxin	21.22745	47.38706	1.158562	4.93E-08	5.39E-06	Upregulated
Ppp1r36	11.22307	25.14689	1.163913	2.55E-05	0.001346	Upregulated
Spag17	12.00798	26.9615	1.166908	1.74E-05	0.000973	Upregulated
Pld5	61.69832	139.9741	1.181857	1.77E-16	7.82E-14	Upregulated
Ccdc121	9.856688	22.38966	1.183658	9.73E-05	0.004328	Upregulated
Adra2b	7.792388	17.81816	1.193211	0.000205	0.0081	Upregulated
Mlf1	11.58809	26.52098	1.194491	9.01E-06	0.000551	Upregulated
Rgs22	15.75539	36.20746	1.200442	6.28E-07	5.37E-05	Upregulated
Rarres2	32.04933	74.10333	1.209244	1.84E-11	3.78E-09	Upregulated
Cd109	19.34967	44.92606	1.215244	2.1E-08	2.39E-06	Upregulated
Ncapg	7.098572	16.51954	1.218573	0.000271	0.010245	Upregulated
1500015O10Rik	20.53301	47.84299	1.220363	4.82E-09	6.46E-07	Upregulated
Slitrk6	9.845869	22.97052	1.222193	3.07E-05	0.001583	Upregulated
Olfm4	6.611726	15.45468	1.224945	0.000231	0.008995	Upregulated
Cdca3	14.14025	33.10585	1.227279	8.8E-07	7.13E-05	Upregulated
2810417H13Rik	14.98822	35.42084	1.240769	3.28E-07	3.09E-05	Upregulated
Vit	26.26238	62.49598	1.250766	9.13E-11	1.74E-08	Upregulated
Kif2c	5.077939	12.10219	1.252953	0.00177	0.048926	Upregulated
Lrrc36	14.07936	33.68619	1.258575	1.12E-06	8.92E-05	Upregulated
1700003D09Rik	17.43571	41.97098	1.267347	1.28E-08	1.5E-06	Upregulated
Cdk1	9.792096	23.75116	1.278308	5.1E-06	0.000327	Upregulated
Dnajb13	5.9575	14.50218	1.283491	0.000533	0.018022	Upregulated

Cdc20	9.520948	23.34379	1.293862	6.45E-06	0.000405	Upregulated
Aurkb	8.596578	21.27399	1.307256	8.03E-06	0.000496	Upregulated
1700026D08Rik	10.84577	27.01589	1.316676	2.02E-06	0.000147	Upregulated
Eppk1	4.4346	11.06126	1.31864	0.002953	0.074056	Upregulated
Pappa	4.927513	12.29074	1.31864	0.000882	0.027527	Upregulated
Rerg	70.04116	176.5992	1.334204	1.4E-21	8.63E-19	Upregulated
Car3	6.259263	15.79196	1.335125	0.000107	0.004664	Upregulated
Cdca2	7.428323	18.74515	1.335409	2.32E-05	0.00125	Upregulated
Crabp1	5.161515	13.10043	1.343748	0.000438	0.015401	Upregulated
Pbk	8.903693	22.72973	1.352105	2.81E-06	0.000193	Upregulated
Tctex1d4	4.184388	10.69292	1.353567	0.00136	0.039871	Upregulated
Spef2	20.11992	51.51913	1.356484	1.22E-10	2.3E-08	Upregulated
Abcc12	4.57245	11.75127	1.361778	0.000813	0.025691	Upregulated
Zic4	79.61724	204.6645	1.362108	1.53E-24	1.15E-21	Upregulated
Odf3b	19.65488	50.59455	1.364094	1.78E-10	3.15E-08	Upregulated
Troap	4.005899	10.31704	1.364831	0.001462	0.042534	Upregulated
Ndst4	24.91965	64.37863	1.369298	2.2E-12	5.2E-10	Upregulated
Cenpf	24.573	63.98106	1.380571	3.53E-13	8.98E-11	Upregulated
Kif18b	6.623642	17.24707	1.380655	4.15E-05	0.002056	Upregulated
Slc14a2	14.80522	38.90772	1.393951	2.5E-09	3.51E-07	Upregulated
Gdnf	4.109979	10.81594	1.395957	0.001125	0.033855	Upregulated
Hdc	16.27798	43.50251	1.418177	2.72E-10	4.7E-08	Upregulated
Bub1	5.347291	14.32316	1.42147	0.000141	0.005955	Upregulated
Crygn	4.738714	12.70798	1.423167	0.000293	0.010958	Upregulated
Hydin	32.43875	87.70671	1.434969	4.76E-16	1.94E-13	Upregulated
Ube2c	7.428544	20.08744	1.435143	3.57E-06	0.000239	Upregulated
Col6a3	56.94825	154.5462	1.440314	2.83E-23	1.86E-20	Upregulated
Nek5	4.382893	12.1391	1.469706	0.000481	0.016493	Upregulated
Wdr93	4.780939	13.31917	1.478138	0.000144	0.006051	Upregulated
Scn4b	691.3522	1966.544	1.50817	4.04E-45	8.23E-42	Upregulated
Capsl	10.86851	31.102	1.516852	8.22E-09	1.05E-06	Upregulated
Lrrc18	4.094636	11.75387	1.521328	0.000229	0.00892	Upregulated
Slc39a4	4.853102	13.94833	1.523113	8.65E-05	0.003952	Upregulated
Mia	7.772864	22.59994	1.539801	2.22E-07	2.21E-05	Upregulated
Prlr	19.12706	55.9854	1.549435	1.45E-14	4.86E-12	Upregulated
Pifo	6.517908	19.21062	1.559423	1.21E-06	9.57E-05	Upregulated
Nexn	22.42494	66.11928	1.559967	4.67E-15	1.7E-12	Upregulated
Gm10714	3.711991	10.94972	1.560629	0.000355	0.012811	Upregulated
Dnah6	42.15396	124.6465	1.564102	1.28E-22	8.12E-20	Upregulated
Spata17	3.486572	10.3672	1.572145	0.000326	0.011998	Upregulated
Musk	11.15017	33.26887	1.577107	1.89E-09	2.75E-07	Upregulated
Cfap126	12.40841	37.21839	1.584697	1.47E-10	2.69E-08	Upregulated
Daw1	6.320066	19.00398	1.58829	1.52E-06	0.000116	Upregulated
Akap14	4.911146	14.81407	1.592837	9.7E-06	0.000585	Upregulated
Rd3	4.461191	13.50854	1.598372	5.24E-05	0.002524	Upregulated
Casc5	5.228315	15.89526	1.604178	8.79E-06	0.000538	Upregulated

Ptprv	15.44565	47.33671	1.615759	2.87E-13	7.49E-11	Upregulated
Esco2	5.13758	15.79695	1.620485	7.35E-06	0.00046	Upregulated
Ccnb1	4.651475	14.56663	1.646907	1E-05	0.000604	Upregulated
Dsc3	4.803076	15.12316	1.654729	1.02E-05	0.00061	Upregulated
Lpar3	3.444501	10.84853	1.655133	0.000169	0.006905	Upregulated
Cfap221	3.149227	10.04955	1.674061	0.000283	0.010612	Upregulated
Phex	7.041565	22.70852	1.689266	1.58E-07	1.63E-05	Upregulated
C230072F16Rik	8.313392	27.25966	1.713259	3.11E-09	4.3E-07	Upregulated
Calml4	4.925864	16.35314	1.731119	1.26E-06	9.85E-05	Upregulated
Scgn	13.06061	43.80534	1.745885	4.83E-14	1.51E-11	Upregulated
1700001C02Rik	3.435145	11.75104	1.774346	4.38E-05	0.002146	Upregulated
Pif1	3.414355	11.80664	1.789913	2.18E-05	0.001185	Upregulated
Nts	16.43063	56.88098	1.791558	4.31E-17	1.99E-14	Upregulated
Dnali1	16.23574	57.16784	1.81603	2.92E-17	1.45E-14	Upregulated
Ccdc146	8.01115	28.73633	1.842795	6.67E-11	1.28E-08	Upregulated
Scn5a	44.46957	163.7994	1.88104	3.64E-36	4.11E-33	Upregulated
Sntn	4.243201	15.98816	1.913779	1E-06	8.04E-05	Upregulated
Clec12a	3.472383	13.10028	1.915599	2.15E-06	0.000154	Upregulated
Nkx2-1	2.874801	10.8767	1.919707	2.18E-05	0.001185	Upregulated
Cxcl5	11.71134	44.58586	1.92868	3.3E-16	1.37E-13	Upregulated
Zfp474	5.674546	21.96781	1.952814	3.16E-09	4.34E-07	Upregulated
Slc35d3	19.48162	76.74026	1.97787	4.6E-24	3.34E-21	Upregulated
Ttll6	4.816383	19.04247	1.983198	1.9E-08	2.17E-06	Upregulated
Car9	7.272977	29.11299	2.001045	4.64E-12	1.07E-09	Upregulated
Dnah11	12.89845	52.02012	2.011872	1.03E-18	5.35E-16	Upregulated
Igfbpl1	33.23171	138.0448	2.054504	3.45E-38	4.12E-35	Upregulated
Ccdc170	4.407203	19.05289	2.112074	2.49E-09	3.51E-07	Upregulated
Ano2	29.34772	131.7397	2.16637	3.41E-40	4.95E-37	Upregulated
1700012B09Rik	2.787801	12.59495	2.175646	5.79E-07	5.03E-05	Upregulated
C130060K24Rik	2.229671	10.27745	2.204579	3.98E-06	0.000264	Upregulated
Mc3r	4.031979	19.1181	2.245379	3.91E-10	6.52E-08	Upregulated
Wdr63	3.096477	17.2292	2.476156	2.89E-10	4.9E-08	Upregulated
Ptpn7	1.980574	11.03256	2.477777	2.4E-07	2.37E-05	Upregulated
Otx2	2.707508	18.07925	2.739298	7.33E-12	1.67E-09	Upregulated
A230065H16Rik	5.394403	55.15992	3.354085	5.32E-39	6.76E-36	Upregulated
Dsg1c	1.104576	14.33857	3.698336	2.36E-13	6.31E-11	Upregulated
Ltf	17.81367	1.618044	-3.46066	8.54E-16	3.34E-13	Downregulated
Ngp	21.12622	2.441284	-3.11332	7.07E-17	3.2E-14	Downregulated
Mmp8	12.69872	2.938817	-2.11138	5.51E-07	4.81E-05	Downregulated
Chil3	18.49271	4.632933	-1.99696	1.12E-08	1.36E-06	Downregulated
S100a8	61.5541	17.91574	-1.78063	1.31E-18	6.65E-16	Downregulated
Retnlg	17.15899	6.438416	-1.41419	2.8E-05	0.001459	Downregulated
S100a9	74.37668	29.51687	-1.33331	1.18E-13	3.32E-11	Downregulated
Sele	33.74156	14.20929	-1.24769	6.64E-08	7.11E-06	Downregulated
Selp	45.48729	19.7219	-1.20567	1.25E-10	2.3E-08	Downregulated
Gh	17.85815	8.153649	-1.13106	0.003648	0.087083	Downregulated

Slfn4	11.96347	5.488176	-1.12424	0.003676	0.087653	Downregulated
Tnfsf8	14.52647	6.872899	-1.07969	0.001284	0.037951	Downregulated
Gm5415	26.59338	13.85774	-0.94038	9.83E-05	0.004354	Downregulated
C7	47.54061	24.88366	-0.93396	2.66E-06	0.000186	Downregulated
Gm10635	105.8168	62.48891	-0.7599	9.88E-07	7.96E-05	Downregulated
Tmem40	64.1732	38.09845	-0.75224	2.41E-05	0.001284	Downregulated
Aoc3	26.26268	15.76077	-0.73668	0.001928	0.052155	Downregulated
Mmp9	28.04716	16.89124	-0.73158	0.002506	0.064355	Downregulated
Pla2g4e	276.0293	166.568	-0.72871	1.01E-09	1.55E-07	Downregulated
Otop2	25.99236	15.8119	-0.71708	0.003173	0.077849	Downregulated
Bcl3	30.95611	18.91684	-0.71055	0.001842	0.050627	Downregulated
Myh1	30.73194	18.83102	-0.70663	0.002143	0.05603	Downregulated
Xlr3b	29.17868	18.41136	-0.66432	0.00415	0.09636	Downregulated
Fap	48.56272	30.83624	-0.65522	0.00081	0.025616	Downregulated
Klhl40	149.0574	96.04701	-0.63406	2.08E-06	0.00015	Downregulated
Socs3	155.2334	100.1539	-0.63222	8.64E-07	7.03E-05	Downregulated
Twist2	36.43812	24.03259	-0.60046	0.003233	0.079124	Downregulated
Hkdc1	376.9012	248.9805	-0.59815	2.41E-07	2.37E-05	Downregulated
AI115009	60.10153	40.1354	-0.58253	0.001135	0.034042	Downregulated
