# Supplementary material for: Transcriptional signatures in prefrontal cortex confer vulnerability versus resilience to food and cocaine addiction-like behavior
Source: Sci Rep. 2021 Apr 27;11:9076. doi: 10.1038/s41598-021-88363-9 (PMC8079697; doi:10.1038/s41598-021-88363-9)
Supplement: Supplementary file 3 — Supplementary Table S3. [file 41598_2021_88363_MOESM3_ESM.pdf]

Transcriptional signatures in prefrontal cortex confer vulnerability versus resilience to food and cocaine addiction-like behavior

Mohit Navandar[1], Elena Martín-García[2], Rafael Maldonado[2,3], Beat Lutz[4,5], Susanne Gerber[1#], Inigo Ruiz de Azua[4,5#]

[1] Institute for Human Genetics, University Medical Center of the Johannes Gutenberg University Mainz, Mainz, Germany

[2] Laboratory of Neuropharmacology-Neurophar, Department of Experimental and Health Sciences, Universitat Pompeu Fabra (UPF), Barcelona, Spain.

[3] Hospital del Mar Medical Research Institute (IMIM), Barcelona, Spain.

[4] Institute of Physiological Chemistry, University Medical Center of the Johannes Gutenberg University Mainz, Mainz, Germany

[5] Leibniz Institute for Resilience Research (LIR), Mainz, Germany

[#] These authors jointly supervised this work

Corresponding author: Inigo Ruiz de Azua. E-mail: Inigo.azua@lir-mainz.de

Supplementary table S3: Details of gene ontologies for 56 shared genes.

GO Term Name	p-value	q-value FDR B&H	Number of genes	Contributing genes
Learning or memory	2.1E-05	0.001685	7	Drd1, Drd2, Ntrk1, Glp1r, Ppp1r1b, Chat, Gpr88
Feeding behavior	2.45E-05	0.001742	5	Adora2a, Drd1, Drd2, Glp1r, Tacr3
Behavioral response to cocaine	4.01E-05	0.002282	3	Drd1, Drd2, Ppp1r1b
Multicellular organismal response to stress	7.1E-06	0.0008037	5	Thbs4, Drd1, Ido1, Ntrk1, Penk
Regulation of long-term synaptic potentiation	5.69E-05	0.002636	3	Adora2a, Drd1, Drd2
Prepulse inhibition	1.21E-05	0.001152	3	Adora2a, Drd1, Drd2
Synaptic transmission, dopaminergic	4.37E-05	0.002317	4	Adora2a, Drd1, Drd2, Rgs9
Regulation of synaptic transmission, glutamatergic	0.000144	0.0049	3	Adora2a, Drd1, Ntrk1
Calcium ion transport	0.000326	0.007205	7	Adora2a, Drd1, Drd2, Cd4, Glp1r, Rgs9, Myb
Histone phosphorylation	0.000194	0.005724	3	Drd1, Ppp1r1b, Prkcd
Regulation of cAMP-mediated signaling	6.1E-06	0.0007234	7	Adora2a, Drd1, Drd2, Galr1, Glp1r, Pde10a, Gpr101
