# Supplementary material for: Transcriptional signatures in prefrontal cortex confer vulnerability versus resilience to food and cocaine addiction-like behavior
Source: Sci Rep. 2021 Apr 27;11:9076. doi: 10.1038/s41598-021-88363-9 (PMC8079697; doi:10.1038/s41598-021-88363-9)

Transcriptional signatures in prefrontal cortex confer vulnerability versus resilience to food and cocaine addiction-like behavior

Mohit Navandar[1], Elena Martín-García[2], Rafael Maldonado[2,3], Beat Lutz[4,5], Susanne Gerber[1#], Inigo Ruiz de Azua[4,5#]

[1] Institute for Human Genetics, University Medical Center of the Johannes Gutenberg University Mainz, Mainz, Germany

[2] Laboratory of Neuropharmacology-Neurophar, Department of Experimental and Health Sciences, Universitat Pompeu Fabra (UPF), Barcelona, Spain.

[3] Hospital del Mar Medical Research Institute (IMIM), Barcelona, Spain.

[4] Institute of Physiological Chemistry, University Medical Center of the Johannes Gutenberg University Mainz, Mainz, Germany

[5] Leibniz Institute for Resilience Research (LIR), Mainz, Germany

[#] These authors jointly supervised this work

Corresponding author: Inigo Ruiz de Azua. E-mail: Inigo.azua@lir-mainz.de

Supplementary Fig 1: Gene expression signature in PFC in addictive-like behaviors in palatable food and cocaine addiction. (a) Boxplot representation of expression of shared genes such as Chat, Glp1r, Pde10a, Penk, Ppp1r1b, Rgs9, Tmem212 and Top2a in non-addicted and addicted mice from both studies **(b)** Boxplot representation of expression of shared genes acting as transcription factors in non-addicted and addicted mice from both studies. **(c)** Volcano plot of RNA-seq data representing the gene expression changes of significantly up-regulated genes (51) and down-regulated genes (74) in cocaine non-addicted mice compared to saline self-administrated mice.

Supplementary Fig 1

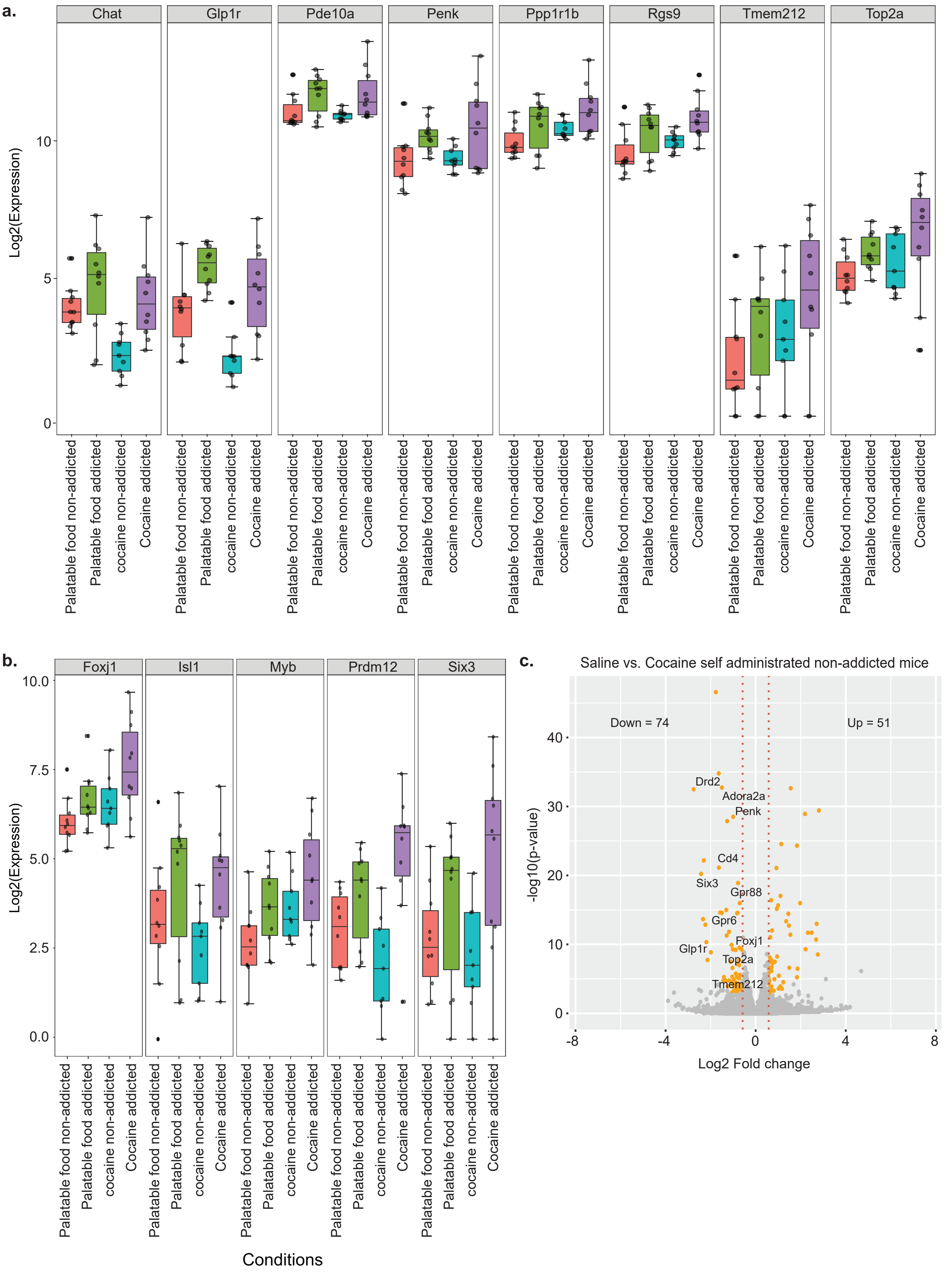

Supplement: Supplementary file 4 — Supplementary Figure 1. [file 41598_2021_88363_MOESM4_ESM.pdf]
