# Supplementary material for: Transcriptional signatures in prefrontal cortex confer vulnerability versus resilience to food and cocaine addiction-like behavior
Source: Sci Rep. 2021 Apr 27;11:9076. doi: 10.1038/s41598-021-88363-9 (PMC8079697; doi:10.1038/s41598-021-88363-9)

Transcriptional signatures in prefrontal cortex confer vulnerability versus resilience to food and cocaine addiction-like behavior

Mohit Navandar[1], Elena Martín-García[2], Rafael Maldonado[2,3], Beat Lutz[4,5], Susanne Gerber[1#], Inigo Ruiz de Azua[4,5#]

[1] Institute for Human Genetics, University Medical Center of the Johannes Gutenberg University Mainz, Mainz, Germany

[2] Laboratory of Neuropharmacology-Neurophar, Department of Experimental and Health Sciences, Universitat Pompeu Fabra (UPF), Barcelona, Spain.

[3] Hospital del Mar Medical Research Institute (IMIM), Barcelona, Spain.

[4] Institute of Physiological Chemistry, University Medical Center of the Johannes Gutenberg University Mainz, Mainz, Germany

[5] Leibniz Institute for Resilience Research (LIR), Mainz, Germany

[#] These authors jointly supervised this work

Corresponding author: Inigo Ruiz de Azua. E-mail: Inigo.azua@lir-mainz.de

Supplementary Fig 4: t-SNE plot representing expression profile of the relevant shared genes in the cell clusters.

Supplementary Fig 4

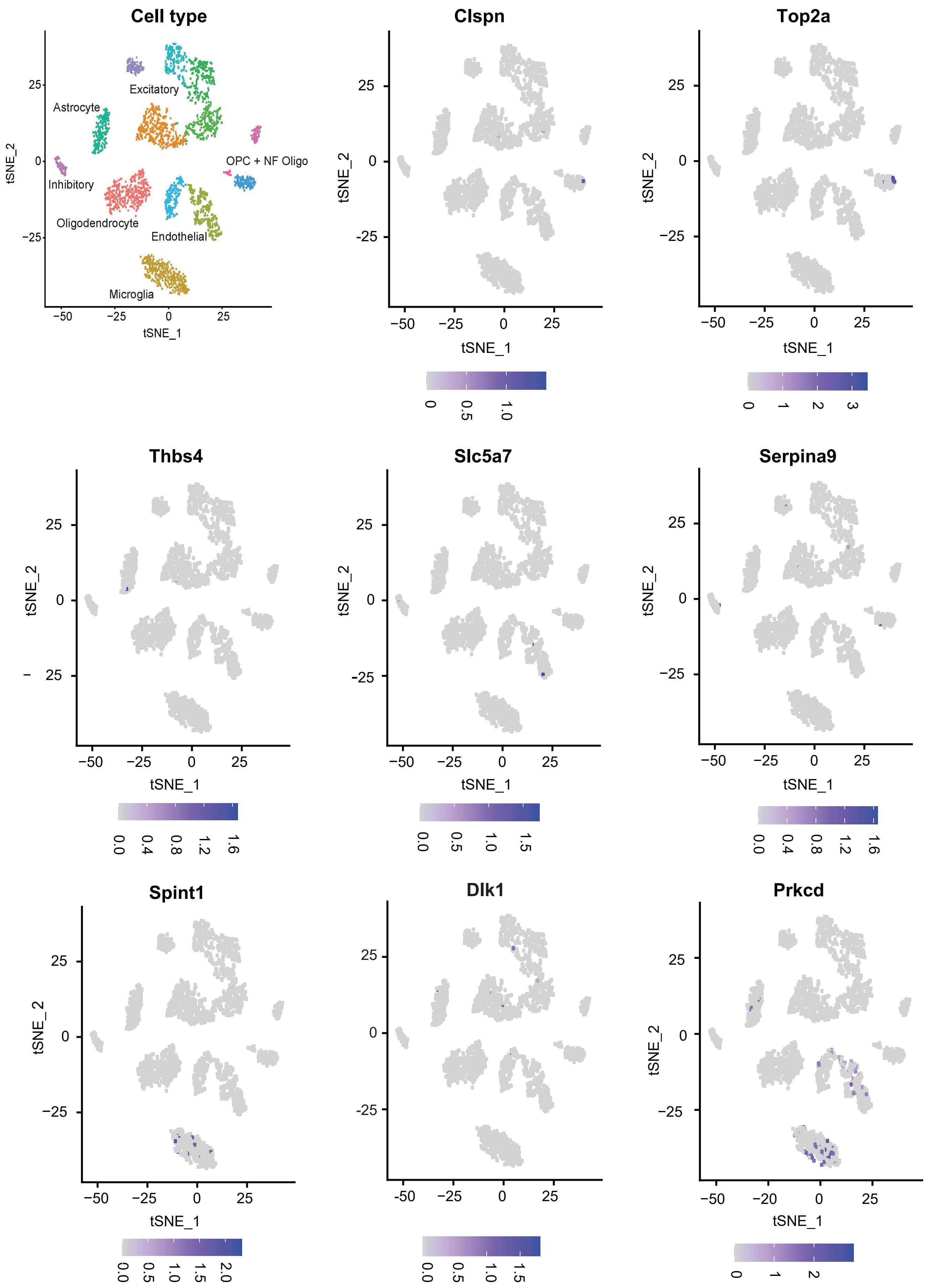

Supplement: Supplementary file 7 — Supplementary Figure 4. [file 41598_2021_88363_MOESM7_ESM.pdf]
